# Neutrophil-microglia interaction drives motor dysfunction in a neuromyelitis optica model induced by subarachnoid AQP4-IgG

**DOI:** 10.1172/JCI199706

**Published:** 2026-02-10

**Authors:** Fangfang Qi, Vanda A. Lennon, Shunyi Zhao, Yong Guo, Husheng Ding, Caiyun Liu, Whitney M. Bartley, Tingjun Chen, Claudia F. Lucchinetti, Long-Jun Wu

**Affiliations:** 1 Neuroimmunology Research Laboratory, Department of Laboratory Medicine and Pathology, and; 2 Department of Neurology, Mayo Clinic, Rochester, Minnesota, USA.; 3 Mayo Clinic Graduate School of Biomedical Sciences, Rochester, Minnesota, USA.; 4 Center for Neuroimmunology and Glial Biology, Institute of Molecular Medicine, McGovern Medical School, University of Texas Health Science Center at Houston, Houston, Texas, USA.; 5 Department of Neurology, Dell Medical School at The University of Texas at Austin, Austin, Texas, USA.

**Keywords:** Autoimmunity, Neuroscience, Autoimmune diseases

## Abstract

Neutrophils and neutrophil extracellular traps (NETs) contribute to early neuromyelitis optica (NMO) histopathology initiated by IgG targeting astrocytic aquaporin-4 (AQP4) water channels. Yet, the mechanisms underlying neutrophil recruitment and their pathogenic roles in disease progression remain unclear. To investigate molecular-cellular events preceding classical complement cascade activation in a mouse NMO model, we continuously infused, via spinal subarachnoid route, a non-complement-activating mouse monoclonal AQP4-IgG. Parenchymal infiltration of netting neutrophils containing C5a ensued with microglial activation and motor impairment but no blood-brain barrier leakage. Motor impairment and neuronal dysfunction both reversed when AQP4-IgG infusion stopped. Two-photon microscopy and electron microscopy–based reconstructions revealed physical interaction of infiltrating neutrophils with microglia. Ablation of either peripheral neutrophils or microglia attenuated the motor deficit, highlighting their synergistic pathogenic roles. Of note, mice lacking complement receptor C5aR1 exhibited reduction in neutrophil infiltration, microglial lysosomal activation, neuronal lipid droplet burden, and motor impairment. Pharmacological inhibition of C5aR1 recapitulated this protection. Immunohistochemical analysis of an NMO patient’s spinal cord revealed disease-associated microglia surrounding motor neurons in nondestructive lesions. Our study identifies neutrophil-derived C5a signaling through microglial C5aR1 as a key early driver of reversible motor neuron dysfunction in the precytolytic phase of NMO.

## Abstract 2

Neutrophil-derived C5a coactivates microglia to drive reversible motor paresis initiated by non-complement-activating aquaporin-4-IgG binding to astrocytes in mouse model of neuromyelitis optica

## Graphical abstract

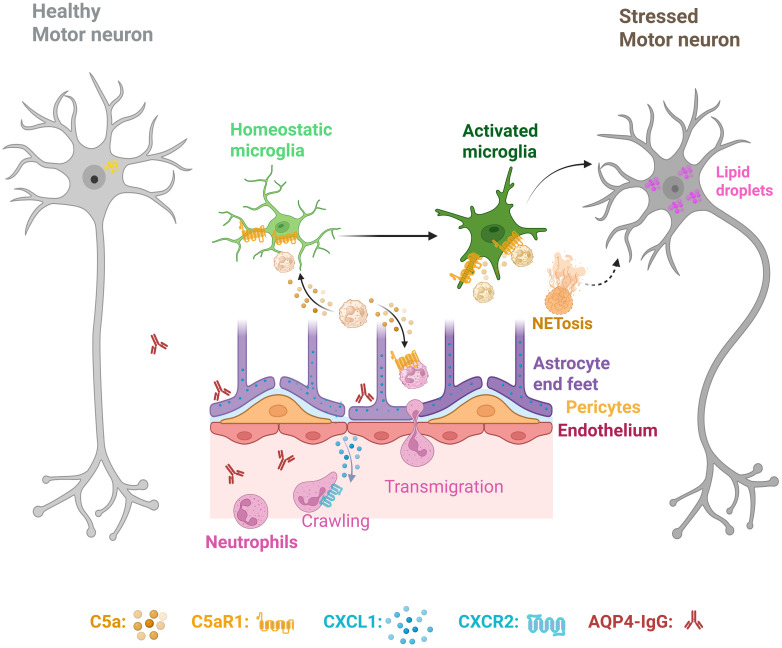

## Introduction

Neuromyelitis optica (NMO) spectrum disorder is a severe, relapsing, inflammatory, autoimmune disorder of the central nervous system (CNS) with myelin loss occurring secondary to astrocyte targeting by a complement-activating IgG specific for the aquaporin-4 (AQP4) water channel (1–3). A C5-neutralizing IgG (eculizumab) is highly effective therapeutically in terminating acute NMO attacks and suppressing relapse (4). Untreated, the fully established NMO lesion shows extensive deposition of the C5b-C9 membrane attack complex on astrocytes, blood-brain barrier (BBB) leakage, and gross tissue destruction (5). These late events in the acute-attack phase require BBB disruption to permit CNS influx of plasma IgG, rheumatoid factor–like IgM (6), and liver-derived complement components.

The presence of neutrophils in early CNS lesions (5, 7, 8) is a histopathological feature distinguishing NMO from other inflammatory, immune-mediated, demyelinating diseases, such as multiple sclerosis, which generally lack neutrophils (9, 10). Neutrophil invasion of the spinal cord (5) and brain parenchyma (7) is thought to contribute to astrocyte dysfunction and tissue damage (11).

Microglia, as CNS immune sentinel cells, continuously monitor and respond to parenchymal changes (12–15). A mouse model of NMO established in this laboratory demonstrated that motor neuronal dysfunction requires microglial activation by complement component C3a emanating from AQP4-IgG–activated astrocytes (16). However, it remains unknown how resident microglia interact with infiltrating neutrophils and influence NMO lesion progression. To investigate early pathogenic events preceding classical complement cascade activation in evolving NMO lesions, we modified our established mouse model (16) by infusing continuously, via spinal subarachnoid route, a non-complement-activating mouse monoclonal IgG1 specific for the AQP4 extracellular domain (clone mECD). Hypothesizing a pathophysiological role for CNS-infiltrating neutrophils in initiating early NMO lesions through chemotaxis (11) and cytokine release (17, 18), we employed high-parameter flow cytometry and electron microscopy to characterize these neutrophils and their interactions. Our findings reveal that physical neutrophil-microglia interaction is a prerequisite for neurological impairment in the precytolytic phase of NMO. Early NMO deficits that in patients are sometimes reversible by plasma exchange and antiinflammatory corticosteroid therapy (19) are driven in the mouse model by neutrophil-derived C5a. Genetic deletion of C5a receptor 1 (C5aR1) significantly reduced neutrophil infiltration, motor neuron stress, and functional motor impairment.

## Results

### Neutrophils infiltrate the spinal parenchyma early in the mouse model of NMO.

**Figure 1:**
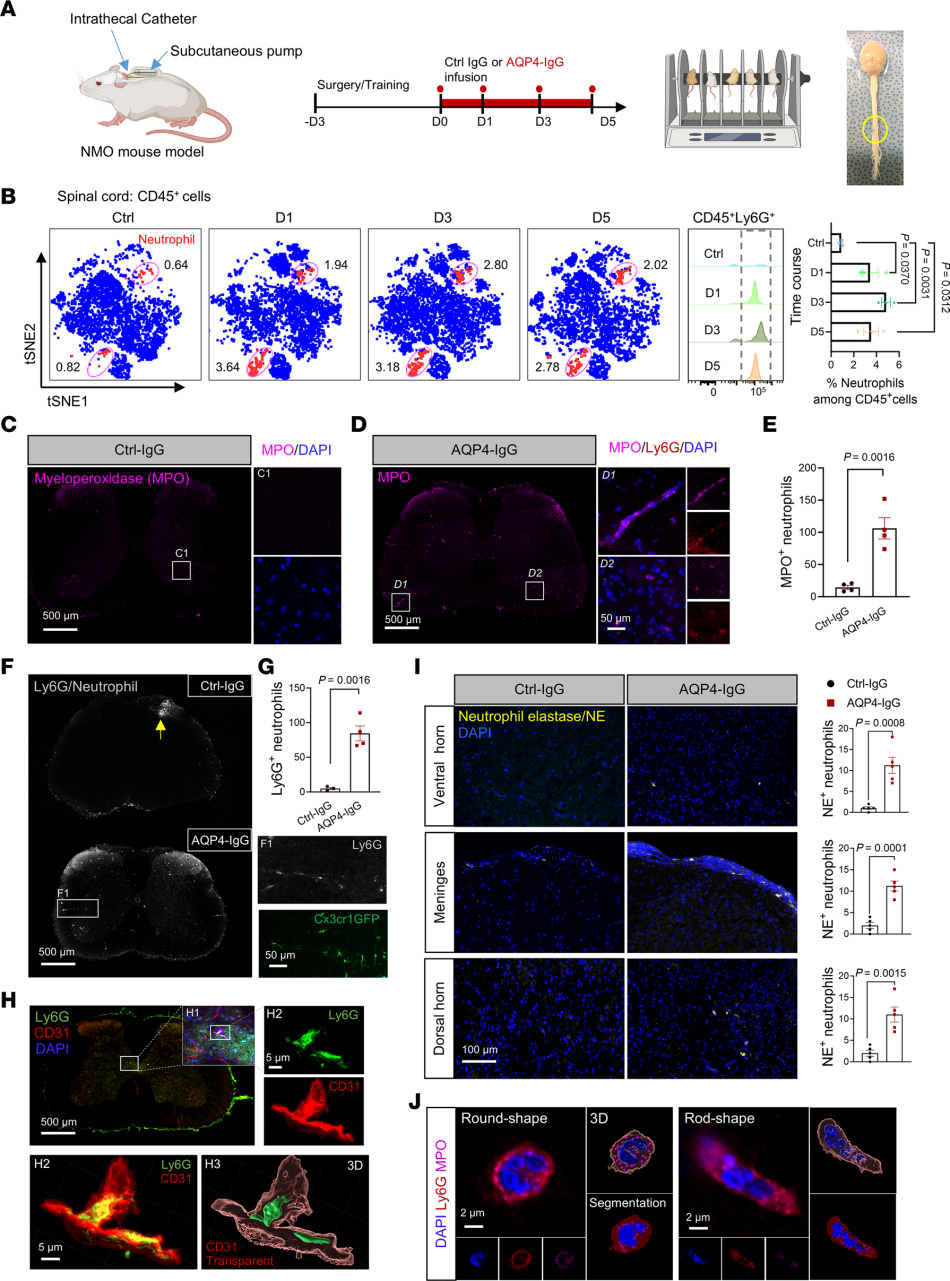
Neutrophils infiltrating and extruding extracellular traps (NETosis) in NMO mouse spinal cord. (**A**) Experimental design: catheter inserted via cisterna magna into L4 subarachnoid space (yellow circle, Evans blue verification). Subcutaneous osmotic pump continuously infuses AQP4-IgG or control mouse IgG (1.2 μg/d, in 12 μL). Rotarod motor training, days –3, –2, –1; testing, days 0, +1, +2, +3, +4, and +5. Terminal transcardiac perfusion: cord harvested for immunohistochemical and flow cytometric analyses. (**B**) Cord-infiltrating neutrophils (Cytek analysis), IgG infusion days 1, 3, and 5. Control mice (first panel) infused 3 days with normal mouse IgG. AQP4-IgG recipients: panels 2–4 (3 mice/group). t-SNE, t-distributed stochastic neighbor embedding. (**C**–**E**) IgG infusion day 3: representative images (**C** and **D**) and quantification (**E**) of neutrophils (Ly6G^+^MPO^+^) in lumbar parenchyma (4 mice/group). Higher magnification box in **C** shows neutrophils (magenta) and nuclei (blue). (**D1** and **D2**) Higher magnification Ly6G^+^MPO^+^ netting neutrophils in AQP4-IgG-infused cord. (**F** and **G**) IgG infusion day 3: representative images in lumbar cord; Ly6G^+^ cluster (top right, control dorsal cord [yellow arrow] is subarachnoid inflammation at catheter site). Higher magnification of **F** shows neutrophils in AQP4-IgG recipient white matter, in or around a blood vessel surrounded by Cx3cr1GFP^+^ microglia/macrophages. Ly6G^+^ neutrophils quantified in cord section; 4 mice/group (**G**). (**H**) Representative images: CD31^+^ blood vessels and Ly6G^+^ neutrophils in AQP4-IgG recipient mouse lumbar cord, day 3. Higher magnification boxed Ly6G^+^/CD31^+^. Higher magnification (split and merged) boxed Ly6G^+^ and CD31^+^. 3D Imaris rendering of Ly6G^+^ neutrophils (green) and CD31^+^ vessel (red; transparent) in **H2**. (**I**) Lumbar cord distribution of NE^+^ neutrophils in ventral horn, meninges, and dorsal horn, day 5 (4 mice/group). (**J**) DAPI^+^ multilobed nucleus (blue) in Ly6G^+^ neutrophil (red, round- and rod-shape) and cytoplasmic MPO^+^ granule protein. 3D rendering and segmentation images show morphology of neutrophil’s nucleus. One-way ANOVA, followed by Tukey’s post hoc multiple comparisons test (**B**). Unpaired *t* test in **E**, **G**, and **I**.

We initially reported microglial activation and motor impairment as outcomes of 5 days’ continuous lumbar subarachnoid infusion of complement-activating serum IgG derived from patients with NMO or a complement-activating monoclonal mouse AQP4-IgG (16). To investigate the contribution of neutrophils in the precytolytic stage of NMO, we infused a non-complement-activating mouse monoclonal IgG1 specific for the AQP4 extracellular domain (Figure 1A). High-spectrum flow cytometry revealed time-dependent infiltration of neutrophils into spinal parenchyma. Numbers peaked on day 3, with CD11b^+^Ly6G^+^Ly6C^neg/lo^ neutrophils accounting for 5.1% of CD45^+^ immune cells; on day 5, 3.9% were neutrophils (Figure 1B and Supplemental Figure 1, A–D; supplemental material available online with this article; https://doi.org/10.1172/JCI199706DS1). Lumbar spinal cord immunostaining on day 3 confirmed neutrophils infiltrating the parenchyma and revealed a greater abundance of neutrophils with neutrophil extracellular traps (NETs; myeloperoxidase [MPO] positive; Figure 1, C–E) as well as Ly6G-positive neutrophils (Figure 1, F and G) in ventral cord gray matter, partly surrounding a blood vessel–like structure (Figure 1, D, D1, and F1). The images of neutrophils within or near blood vessels in the spinal parenchyma of AQP4-IgG recipient mice are consistent with transvascular infiltration (Figure 1H). On day 3, neutrophil elastase–positive (NE-positive) neutrophils were scattered in the ventral horn, dorsal horn, and meninges (Figure 1I); infiltrating NET^+^ neutrophils were 5- to 6-fold more numerous in AQP4-IgG recipients than in control-IgG recipients (Figure 1, E and I). Morphologically, infiltrating neutrophils at high magnification were round shaped and rod shaped with a characteristic multilobed nucleus and contained the cytoplasmic neutrophil–typical MPO granule protein (Figure 1J and Supplemental Figure 1, E and F). Quantitative 3D rendering revealed that NET structures displayed various sizes, with surface areas ranging from a few to several hundred square micrometers and corresponding volumes spanning a similar order of magnitude (Supplemental Figure 2, A–D). Correlations between NET area or volume and sphericity suggested that NET morphology changes could be engaged in the evolution of NMO (Supplemental Figure 2, E and F). These findings are consistent with infiltrating neutrophils being preactivated, still maintaining intact neutrophil morphology at late-stage activation but with granule protein and DNA extrusion evident as tiny web-like structures. Our results indicate that AQP4-IgG infusion into the lumbar subarachnoid space of mice triggers neutrophil migration into the neighboring cord parenchyma, with a subset extruding Neutrophil Extracellular Traps (i.e., undergoing NETosis). Thus, our model closely resembles early-stage CNS lesions observed in patients with NMO (8).

### Neutrophils interact with microglia in NMO.

**Figure 2:**
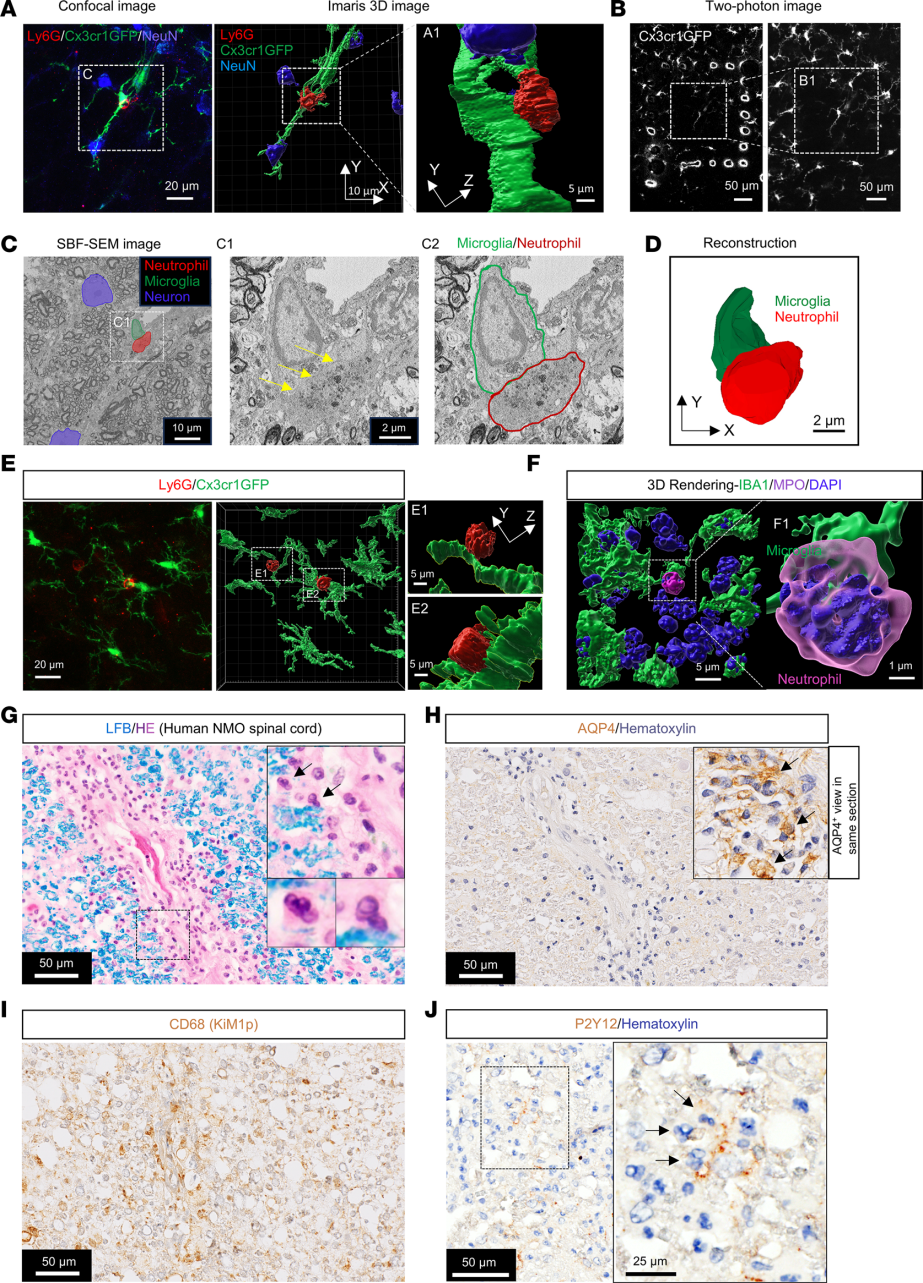
Neutrophil-microglial contacts in lumbar parenchyma, AQP4-IgG infusion day 3. (**A**) Confocal image identifies putatively interacting microglia (Cx3cr1GFP^+^, green) and neutrophil (Ly6G^+^, red) adjacent to a neuronal soma (NeuN^+^, blue); subsequent images are Imaris 3D rendering. (**B**) Cord region of interest containing contacting neutrophil-microglia; laser-branded frame by 2-photon imaging; magnified. (**C**) Serial block-face scanning electron microscopy (SBF-SEM) shows ultrastructurally the same neutrophil, microglia, and 2 neurons boxed in **A**. Yellow arrows (magnified, **C1**) indicate contacting microglial-neutrophil somata edges (green and red lines in **C2**). (**D**) 3D serial reconstruction of contacting microglial-neutrophil somata in **C** (Supplemental Video 1, *Z*-stack). (**E**) Representative confocal image (left, 2,048 × 2,048 pixel; ×63 objective lens) and 3D rendering image (right) show microglial process and soma interacting with Ly6G^+^ neutrophils in lumbar parenchyma of AQP4-IgG recipient (Supplemental Video 2, Imaris). (**F**) Representative 3D rendering image shows netting (MPO^+^) neutrophil interacting with microglial processes in lumbar parenchyma of AQP4-IgG-recipient. High power shows microglial process-neutrophil soma physical interaction (Supplemental Video 3, Imaris). Multilobed nucleus, DAPI^+^ (blue). IBA1, ionized calcium-binding adapter molecule 1. (**G**–**I**) Serial histopathologic sections of NMO patient’s early spinal cord lesion. (**G**) White matter parenchyma shows intact myelin (Luxol fast blue) with infiltrating neutrophils and eosinophils close to a penetrating vessel. Neutrophil infiltration area, right upper inset; 2 infiltrated neutrophils in right lower inset have multilobed nuclei (arrow). (**H**) AQP4 immunoreactivity is reduced in this region; the adjacent area (right inset) retained AQP4. (**I**) CD68 (KiM1p) immunostain identifies macrophages/microglia in this non-demyelinated lesion. (**J**) Neutrophils among P2Y12^+^ microglia; magnification (inset box right) shows 3 neutrophils (segmented nuclei, hematoxylin-stained) abutting microglial processes. Arrows indicate neutrophils contacting microglia.

Microglia potentially respond to local cues emanating from many cell types in evolving CNS lesions induced by subarachnoid AQP4-IgG infusion (16, 20–22). Lumbar cord confocal images of mice with genetically GFP-tagged microglia (Cx3cr1GFP) revealed Ly6G-immunostained neutrophils in proximity to microglia (Figure 2A, left). Imaris 3D rendering of those images revealed potential foci of neutrophil-microglia physical interaction (Figure 2A, middle and right). Near-infrared branding by 2-photon microscopy (Figure 2B) allowed electron microscopy (EM) acquisition of higher resolution images (Figure 2C and Supplemental Figure 3, A–C). Serial block-face scanning electron microscopy enabled the cells of interest observed in confocal images to be identified: microglia (green), neutrophil (red), and 2 neurons (blue) (Figure 2C and Supplemental Video 1). 3D reconstruction based on serial EM images and confocal images confirmed 2 types of physical neutrophil-microglial interactions (Figure 2, C–F): neutrophil soma-microglial process and neutrophil soma-microglial soma. The percentage of each neutrophil interaction type (Ly6G^+^, MPO^+^, DAPI^+^ nucleus multilobed) was enumerated by employing high-resolution *Z*-stacked confocal images and subsequent 3D rendering (Figure 2, E and F, and Supplemental Videos 2 and 3): 47% contacted microglial processes (Figure 2, E1 and F) and 19% contacted microglial somata (Figure 2E2); approximately 34% had no microglial contact (Supplemental Figure 3D).

To investigate the pertinence of these findings to lesion evolution in patients with NMO, we immunohistochemically analyzed early lesions in the spinal cord of a patient (NMO case 1). In white matter (Figure 2G), myelin remained intact, and a penetrating blood vessel was surrounded by abundant granulocytes (predominantly neutrophils). AQP4 immunoreactivity in that region was reduced (Figure 2H) by comparison with an adjacent region in the same section, and microglial/macrophage activation was prominent (Figure 2I). Consistent with findings in the mouse model, we observed P2Y12^+^ microglia interacting with neutrophils in the early pre-demyelinating lesion of this patient’s spinal white matter (Figure 2J). We next assessed BBB integrity in the mouse model by evaluating, on days 3 and 5 of AQP4-IgG infusion, the distribution of endogenous fibrinogen (340 kDa) and dextran red (70 kDa) injected 40 minutes prior to perfusion and of the immunostained endothelial tight junction protein, Claudin-5 (Supplemental Figure 4, A–C). No BBB leakage was evident (Supplemental Figure 4D). Thus, the prominent neutrophil infiltration is not due to BBB compromise. Documentation of CNS-infiltrating neutrophils interacting physically with microglia in early spinal cord lesions of AQP4-IgG–infused mice and in a patient with acute NMO supports a pathogenic role for neutrophils in early-stage NMO lesions.

### Neutrophils drive microglial activation and motor impairment initiated by AQP4-IgG binding to astrocytes.

**Figure 3:**
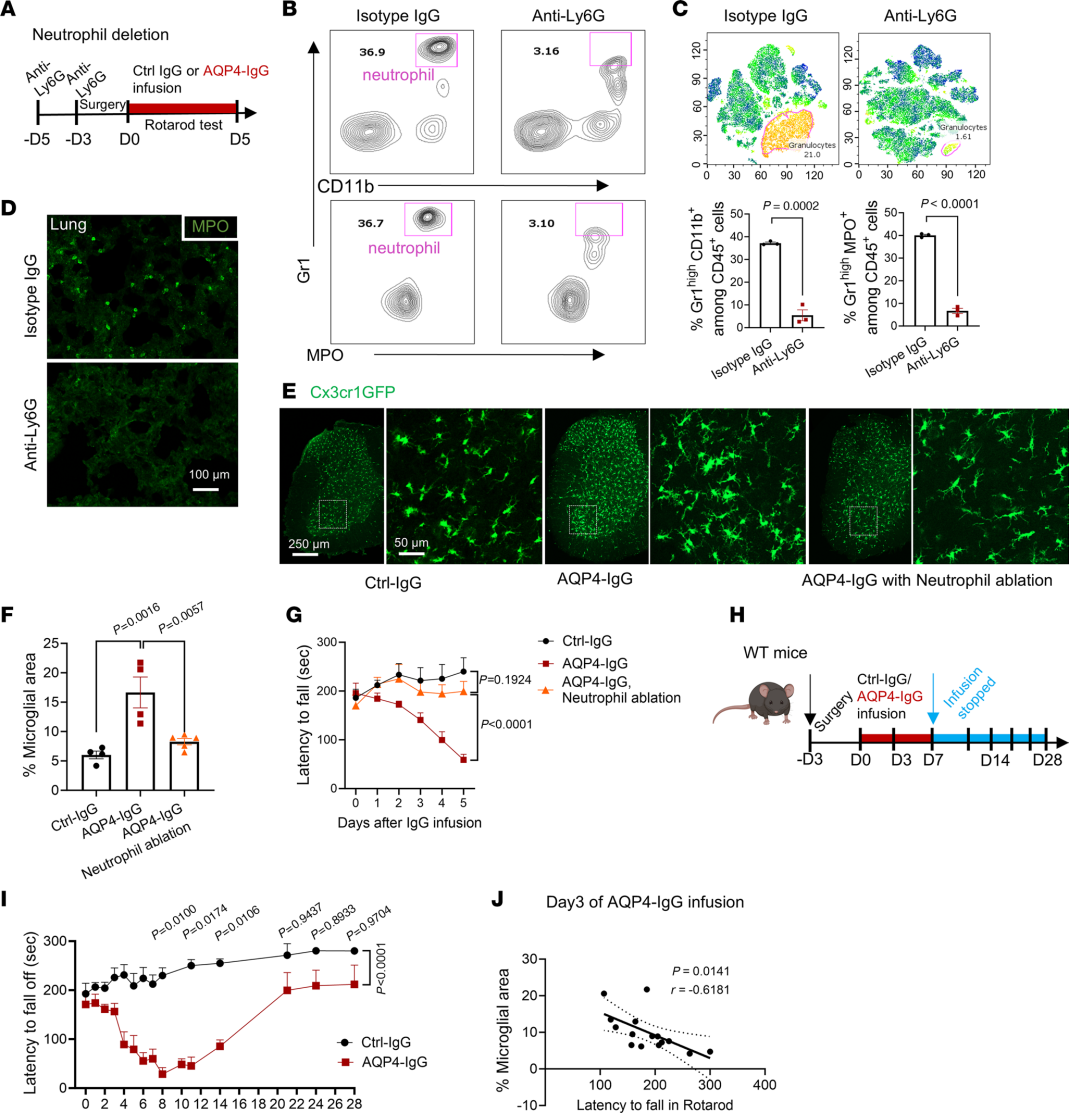
Microglial activation and motor impairment by AQP4-IgG requires CNS-infiltrating neutrophils. (**A**) Timeline for injecting neutrophil-depleting anti-Ly6G-IgG or isotype control-IgG (100 mg/kg, i.p.), inserting lumbar subarachnoid catheter, infusing AQP4-IgG, and rotarod testing. (**B**) Flow cytometry confirms neutrophil ablation efficiency (percentage CD45^+^CD11b^+^Gr1^+^MPO^+^ cells among peripheral CD45^+^CD11b^+^ cells). (**C**) (Upper) t-SNE analysis of CD45^+^ immune cell subtypes from lumbar spinal cords of control and neutrophil-depleted mice. (Lower) Quantification of data in **B** (3 mice/group). (**D**) Representative confocal images of neutrophils in lungs of mice receiving neutrophil-depleting anti-Ly6G-IgG or isotype control-IgG (3 mice/group). (**E**) Microglial activation, reflected by Cx3cr1GFP signal, in corresponding lumbar cord regions of mice without and with neutrophil ablation (by Ly6G-IgG or isotype control-IgG) after 3 days’ infusion with normal control mouse IgG or AQP4-IgG. (**F**) Quantification of microglia-occupied areas in **E** (*n* = 4–5 mice per group). (**G**) Motor function, reflected by rotarod test, in neutropenic mice (anti-Ly6G-IgG–treated) and non-neutrophil-ablated (isotype control-IgG–treated) during 5 days’ infusion of AQP4-IgG or normal control mouse IgG (0.1 μg/μL; time: *F*_(2.415,__28.98)_ = 4.838, *P* = 0.0113; treatment: *F*_(2,__12)_ = 12.46, *P* = 0.0012; interaction: *F*_(10, 60)_ = 7.100, *P* < 0.0001; *n* = 5 mice per group). (**H**) Experimental design: WT mice were continuously infused with Ctrl-IgG or AQP4-IgG by osmotic pumps for 7 days from day 0; infusion was discontinued at day 8. (**I**) Motor impairment worsened progressively in AQP4-IgG recipients, with nadir at day 8. Continued rotarod testing for another 3 weeks showed progressive motor recovery from day 8. (**J**) Correlations between microglial activation state (lumbar microglial area) and latency to fall in rotarod test. Simple linear regression (1 dot represents 1 mouse at day 3 of IgG infusion). Statistics: **C** used *t* test; Tukey’s post hoc multiple comparisons test (1-way ANOVA) in **F**; 2-way repeated measures ANOVA with Holm-Šídák post hoc test in **G** and **I**.

To investigate potential roles of infiltrating neutrophils in neuropathology and motor dysfunction induced by AQP4-IgG, we depleted mature neutrophils by twice injecting Ly6G-specific IgG (100 mg/kg i.p.), 2 days before subarachnoid catheter insertion (Figure 3A and Supplemental Figure 5A). Flow cytometry confirmed loss of circulating neutrophils (Figure 3B); the proportion of mature neutrophils (Gr1^hi^CD11b^+^, Gr1^hi^MPO^+^) among total circulating myeloid cells (CD45^+^CD11b^+^) was significantly reduced in anti-Ly6G-IgG–treated mice compared with isotype control-IgG–treated mice (Figure 3B and t-SNE heatmap in Figure 3C). Conversely, the fraction of immature myeloid cells (Gr1^int^CD11b^+^) and neutrophils (Gr1^int^MPO^+^) was compensatorily increased in mice depleted of mature neutrophils (Supplemental Figure 5, B–E). Similarly, peripheral resident MPO^+^ (NET) cells were rarely detected in the lung sections of anti-Ly6G-IgG–treated mice (Figure 3D). Mice treated with isotype control-IgG exhibited worsening motor impairment and morphologic evidence of microglial activation in lumbar cord parenchyma during AQP4-IgG infusion; neither outcome was observed in neutrophil-depleted recipients of AQP4-IgG (Figure 3, E–G, and Supplemental Video 4). The peak of neutrophil infiltration (day 3 of AQP4-IgG infusion) coincided with morphologically evident microglial activation but preceded peak motor impairment (Figure 3G), which continued beyond day 5 but reversed spontaneously over the course of 3 weeks after stopping AQP4-IgG infusion at day 7 (Figure 3, H and I). The significant correlation between rotarod latency to fall and microglial area expansion (Figure 3J) suggests that microglial activity influences motor impairment severity. Indeed, microglial ablation did mitigate motor impairment (Supplemental Figure 6), consistent with our earlier report (16). These data indicate that, in NMO lesion evolution, infiltrating neutrophils or their secreted products enhance microglial activation and the motor impairment initiated by AQP4-IgG binding to astrocytes.

### Neutrophil-derived C5a enhances NETosis and microglial activation to initiate motor impairment.

**Figure 4:**
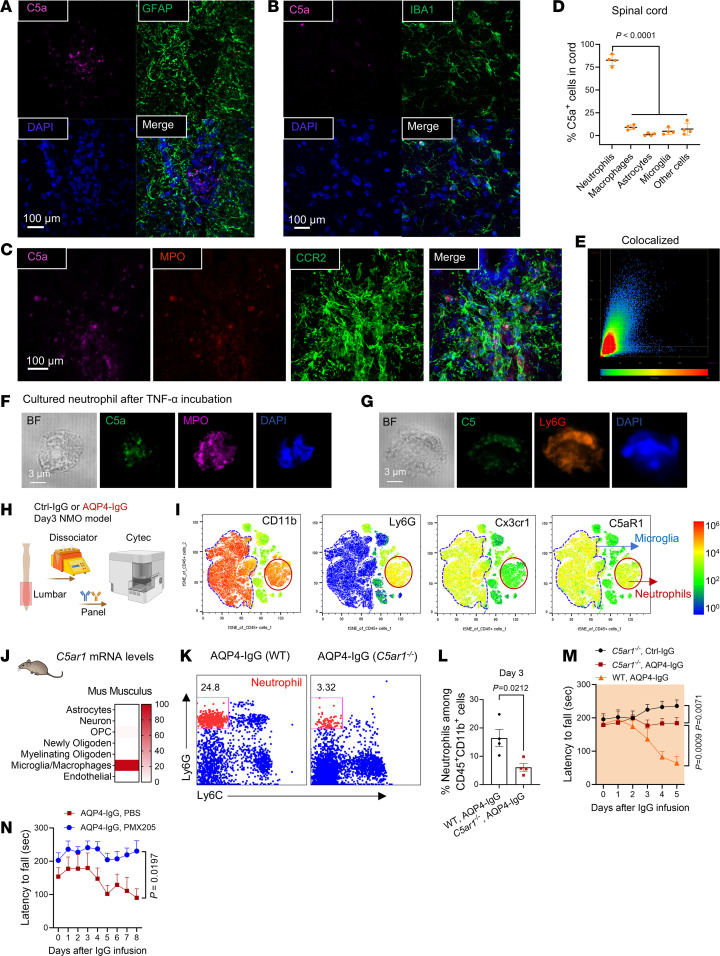
Neutrophil-derived C5a enhances NET production and activates microglia via C5aR1 signaling. (**A** and **B**) Gray matter of AQP4-IgG recipient mice, C5a immunoreactivity was not seen in GFAP^+^ astrocytes (**A**) and rarely seen in IBA1^+^ microglia/macrophages (**B**). (**C**) Triple immunostaining of lumbar cord of AQP4-IgG recipient mice revealed C5a in neutrophils (MPO^+^, left) and monocytes (CCR2-GFP^+^, enhanced by anti-GFP-IgG, right). (**D**) The percentage of spinal cord cells expressing C5a in AQP4-IgG-infused mice (*n* = 4 mice per group). (**E**) Colocalization analysis of C5a and MPO signals in **C** using Zen software. (**F** and **G**) Confocal images of neutrophil (Ly6G, red) containing C5a (green in **F**), and C5 (green in **G**) after TNF-α incubation in vitro. Granule protein, MPO^+^, magenta; nuclear segmentation, DAPI^+^, blue. BF, bright-field. (**H**) Lumbar cords from IgG-infused mice were dissociated enzymatically and subjected to high parametric flow cytometric analysis. (**I**) t-SNE maps identifying CD11b^+^, Ly6G^+^, Cx3cr1^+^, and C5aR1^+^ cells and their expression levels among CD45^+^ immune cells. (**J**) Public database (*Brain RNA-Seq*) documents that *C5ar1* mRNA in normal mouse brain is predominantly expressed in microglia/macrophages. (**K**) Flow cytometric plot shows that neutrophils infiltrate the lumbar cord in WT mice and in *C5ar1-*deficient mice at day 3. (**L**) Quantification of the percentage of neutrophils (CD45^+^CD11b^+^Ly6G^+^Ly6C^–^ cells among CD45^+^CD11b^+^ cells) in **K** (*n* = 4 mice per group). (**M**) Motor function, assessed by rotarod performance (latency to fall), in WT and *C5ar1^–/–^* mice infused with AQP4-IgG or (only *C5ar1^–/–^* mice) normal mouse IgG (0.1 μg/μL, *n* = 6 mice per group). (**N**) Motor function of mice assessed as rotarod fall latency; treatment: *F*_(1,__6)_ = 9.961, *P* = 0.0197; *n* = 6–7 mice per group. Statistics: **D** used Tukey’s post hoc multiple comparisons test in (1-way ANOVA); *t* test in **J**; 2-way repeated measures ANOVA with Holm-Šídák post hoc test in **M** and **N**.

Levels of C5a, the chemoattractant polypeptide cleavage product of complement C5 protein that recruits and activates neutrophils (23, 24), are elevated in cerebrospinal fluid (CSF) of patients with established NMO, in both attack and remission stages (25). We initially assumed that C5 protein was produced and secreted by AQP4-IgG–activated astrocytes as the source of C5a promoting early CNS infiltration and maturation of neutrophils (26). However, we did not detect C5 proenzyme immunoreactivity in any spinal parenchymal cell of mice infused 5 days with AQP4-IgG (Supplemental Figure 7). Further, we found no C5a immunoreactivity in astrocytes (Figure 4A), and very few IBA1^+^ macrophages/microglia or infiltrating CCR2^+^ monocytes were C5a positive (Figure 4, B and C). Instead, the cytoplasm of infiltrating MPO^+^ neutrophils accounted for more than 90% of C5a immunoreactivity (Figure 4, C–E). Thus, activated neutrophils appear to be the initial source of C5a in the evolving spinal cord lesion of this mouse NMO model. To support this contention, we analyzed C5a and C5 in cultured neutrophils stimulated by TNF-α. In agreement with the CNS immunohistochemical findings, cytoplasmic C5a was prominent, and in some neutrophils, C5 holoenzyme was detected after TNF-α stimulation (Figure 4, F and G).

We next evaluated C5aR1 expression on subsets of leukocytes enzymatically dissociated from spinal cord tissue of IgG-infused mice (Figure 4H; gating strategy, Supplemental Figure 1). Microglia and neutrophils highly expressed C5aR1; monocytes and lymphocytes expressed low levels (Figure 4I). These data accord with a public transcriptomic database showing high *C5ar1* mRNA expression in microglia/macrophages of unperturbed mouse CNS tissue (Figure 4J, *Brain RNA-Seq*). The scarcity of lumbar cord–infiltrating neutrophils on day 3 in *C5ar1*^–/–^ mice (Figure 4, K and L) implicates C5a/C5aR1 signaling as the mediator of neutrophil infiltration and subsequent neutrophil-microglial interaction. Consistent with our observations in neutrophil-immunodepleted mice (Figure 3G), genetic deletion of *C5ar1* or pharmacological inhibition of its function by PMX 205 (10 mg/kg) (Figure 4, M and N) mitigated motor impairment.

**Figure 5:**
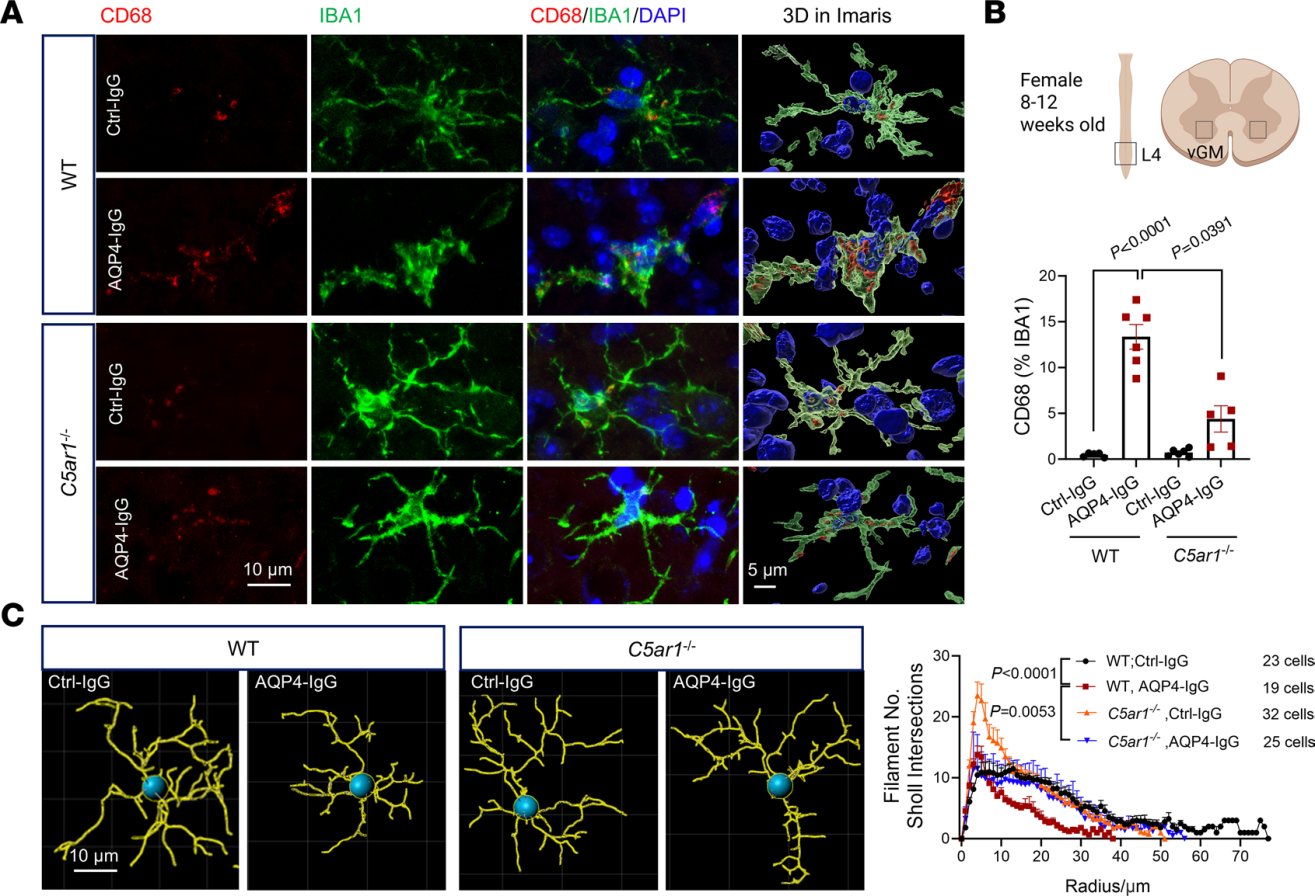
*C5ar1* deficiency abrogates downstream microglial activation response to AQP4-IgG infusion. (**A**) Representative confocal images from lumbar cord of wild-type (WT) and *C5ar1*^-/-^ mice infused with normal IgG or AQP4-IgG. Lysosomal CD68 immunoreactivity (red) is more abundant in microglia (green) of WT recipients of AQP4-IgG than in *C5ar1^–/–^* recipients. Imaris 3D rendering images illustrate the magnitude of lysosomal expansion. (**B**) ImageJ (NIH) analysis of the percentage area occupied by lysosome inside microglia in the lumbar cord of different experimental groups; treatment: *F*_(1,__20)_ = 51.35, *P* < 0.0001; genotype: *F*_(1,__20)_ = 14.17, *P* = 0.0066; *n* = 5–6 mice per group. (**C**) Sholl analysis of microglial branching revealed by Imaris AI-powered filament tracing, which counts the number of microglial filaments intersected by 1 μm spherical steps. Treatment: *F*_(77,__3,730)_ = 39.35, *P* < 0.0001; radius: *F*_(3,__95)_ = 17.02, *P* < 0.0001; *n* = 19–32 microglia from 5 mice per group. Two-way (treatment × genotyping) ANOVA with Holm-Šídák post hoc multiple comparisons test in **B**. Two-way repeated measures ANOVA with Holm-Šídák post hoc test in **C**.

Spinal gray matter is inflamed in patients with early-stage NMO (27–30). To investigate the functional outcome for microglia activated by C5aR1 signaling in this mouse model, we evaluated microglial lysosomal activity in the lumbar ventral horn at the peak of neutrophil infiltration (day 3 of AQP4-IgG infusion). Application of masking and 3D Imaris rendering at the single-cell level revealed prominent CD68^+^ phagolysosome structures within expanded microglial cytoplasm (Figure 5A). By contrast, CD68^+^ lysosome expansion was not detected in *C5ar1*^–/–^ mice (Figure 5A). The morphological alterations in spinal microglia on day 3 (quantitated in Figure 5B) presumably reflect their response to cues promoting lesion progression (31). Analysis by Imaris 10 AI-powered filament tracer revealed morphological alterations in the early response of WT mice to subarachnoid AQP4-IgG infusion, namely shorter microglial processes with less branch complexity (Figure 5C). The reduced complexity of processes in ventral gray matter microglia in the early-stage NMO lesion coincided with phagolysosomal expansion. Neither change was observed in microglia of AQP4-IgG–infused *C5ar1*^–/–^ mice. Together, these results reveal that the C5a/C5aR1 signaling pathway is a critical contributor to microglial activation in this model of NMO.

### Lack of C5aR1 alleviates motor neuronal oxidative stress and lipid droplet accumulation.

**Figure 6:**
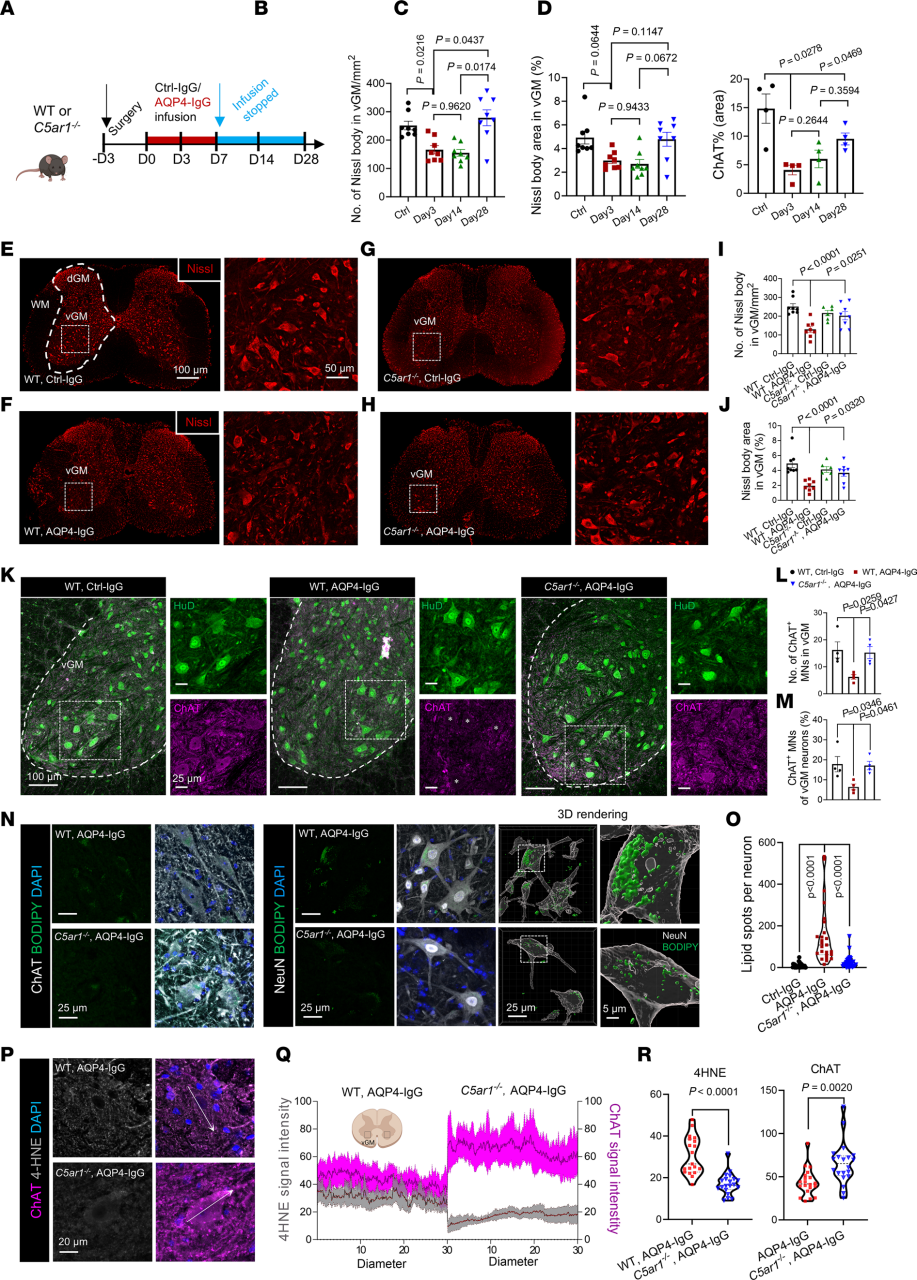
*C5ar1* deficiency ameliorates neuronal dysfunction and lipid droplet accumulation in motor neurons. (**A**) Experimental design: mice were infused continuously with AQP4-IgG using osmotic pumps from day 0 through day 7. (**B**–**D**) Quantification of Nissl bodies (**B**), Nissl area (**C**), and percentage area occupied by ChAT^+^ motor neurons in gray matter of lumbar cord of Ctrl-IgG–infused mice at day 3, AQP4-IgG–infused mice at days 3, 14, and 28. (**E**–**H**) Representative images of Nissl bodies (red) in ventral gray matter (vGM) neurons of wild-type and *C5ar1^–/–^* mice at day 3. Boxed areas are enlarged on the right. (**I** and **J**) Numbers and sizes of Nissl body^+^ neurons in vGM (*n* = 8 mice per group). (**K**) Representative motor neuron confocal images and quantification in vGM of WT mice infused with Ctrl-IgG or AQP4-IgG and *C5ar1^–/–^* mice infused with AQP4-IgG or control-IgG (not shown). HuD^+^ (green); ChAT^+^ (magenta). (**L** and **M**) Numbers of ChAT^+^ motor neurons (MNs) and their percentage among total neurons (HuD^+^ in vGM) (*n* = 4 mice per group). (**N**) Representative images of BODIPY^+^ lipid droplets (green) in ChAT^+^ motor neurons (gray) and NeuN^+^ neurons (gray) in vGM of WT and *C5ar1^–/–^* mice infused with AQP4-IgG. 3D reconstruction of BODIPY^+^ lipid and NeuN^+^ neurons (right). (**O**) Quantification of lipid droplet numbers in the cytoplasm of NeuN^+^ neurons. (**P**) Representative images of 4-HNE (peroxidative stress marker) in ChAT^+^ motor neurons. (**Q** and **R**) **Q**, Quantification of 4-HNE and ChAT immunoreactivity intensities across 30 μm neuronal diameter; **R**, presented as mean ± SEM (*n* = 20 neurons from 4 mice per group). One-way ANOVA with Tukey’s post hoc in **B**–**D**, **L**, **M**, and **O**; 2-way (treatment × genotyping) ANOVA with Holm-Šídák post hoc multiple comparisons test in **I** and **J**; *t* test in **R**.

Weakness or paralysis in NMO attacks (32), and in rodent NMO models induced by intrathecal AQP4-IgG infusion (16, 33, 34), reflects functional impairment of spinal motor neurons. To better understand the pathophysiologic events linking dysfunction of motor neurons to initial astrocyte activation and secondary activation of neutrophils and microglia, we evaluated cytoplasmic Nissl body staining and immunoreactivities of cytoplasmic choline acetyltransferase (ChAT) and nuclear-cytoplasmic HuD proteins as indices of neuronal health during AQP4-IgG infusion. Nissl body and ChAT staining loss was observed in motor neurons on day 3, but consistent with the behavioral data (Figure 3I and Supplemental Figure 8), losses were transient and normalized by day 28 after stopping IgG infusion on day 7 (Figure 6, A–D).

On day 3, Nissl bodies in ventral gray matter neurons exhibited a 41% reduction in both number and average area in AQP4-IgG–infused mice (Figure 6, E, F, I, and J); the number of ChAT^+^ neurons (exclusively motor neurons) was 75% reduced, and the percentage of ChAT^+^/HuD^+^ neurons was 70% reduced (Figure 6, K–M). However, total numbers of HuD^+^ neurons in ventral gray matter did not differ significantly in control-IgG and AQP4-IgG recipient mice (Supplemental Figure 9, A–D). AQP4-IgG recipient mice lacking C5aR1 signaling did not exhibit Nissl body loss (Figure 6, G–J). These observations implicate neutrophil-microglial signaling through C5aR1 in the mediation of motor neuron dysfunction in this mouse NMO model. We next evaluated large ChAT-containing ventral horn neurons for morphologic evidence of dysfunction downstream of AQP4-IgG binding to astrocytes. The 75% reduction in numbers of motor neurons expressing ChAT suggests that the observed Nissl body loss reflects impaired motor neuron function, rather than actual neuronal loss. In mice with genetically ablated C5aR1 signaling, motor neuronal ChAT immunoreactivity was retained at an apparently critical early stage of NMO evolution. The lack of significant difference between WT mice receiving control-IgG or AQP4-IgG in numbers of ventral horn interneurons (HuD^+^/ChAT^–^) (Supplemental Figure 9E) implies an inherent susceptibility of spinal motor neurons (HuD^+^/ChAT^+^) to functional impairment in NMO.

We investigated next whether disruption of astrocyte function, prior to terminal immune-mediated lysis, might impact neuronal lipid metabolism. Application of the neutral lipid stain BODIPY 493 revealed early accumulation of cytosolic lipid droplets in motor neurons during AQP4-IgG infusion (Figure 6, N and O). Significantly fewer lipid droplets were found in corresponding neurons of control-IgG recipient mice (Figure 6O). To determine whether lipid droplet accumulation paralleled lipid peroxidation in motor neurons, we immunostained for the oxidative stress product, 4-hydroxynonenal (4-HNE). Its enhancement coincided with lipid droplet accumulation (*P* < 0.0001 for 4-HNE; *P* = 0.0020 for ChAT; Figure 6, P–R). Importantly, lipid peroxidation and droplet accumulation in spinal motor neurons did not differ significantly in *C5ar1*-deficient recipients of AQP4-IgG and WT recipients of control-IgG. Thus, neuronal lipid droplet accumulation and lipid oxidation in neurons is a C5aR1 signaling–dependent pathophysiological phenomenon occurring early in the evolving NMO lesion, independent of cytolytic complement lesioning.

### Microglia are required for AQP4-IgG to induce astrocytic production of CXCL1.

**Figure 7:**
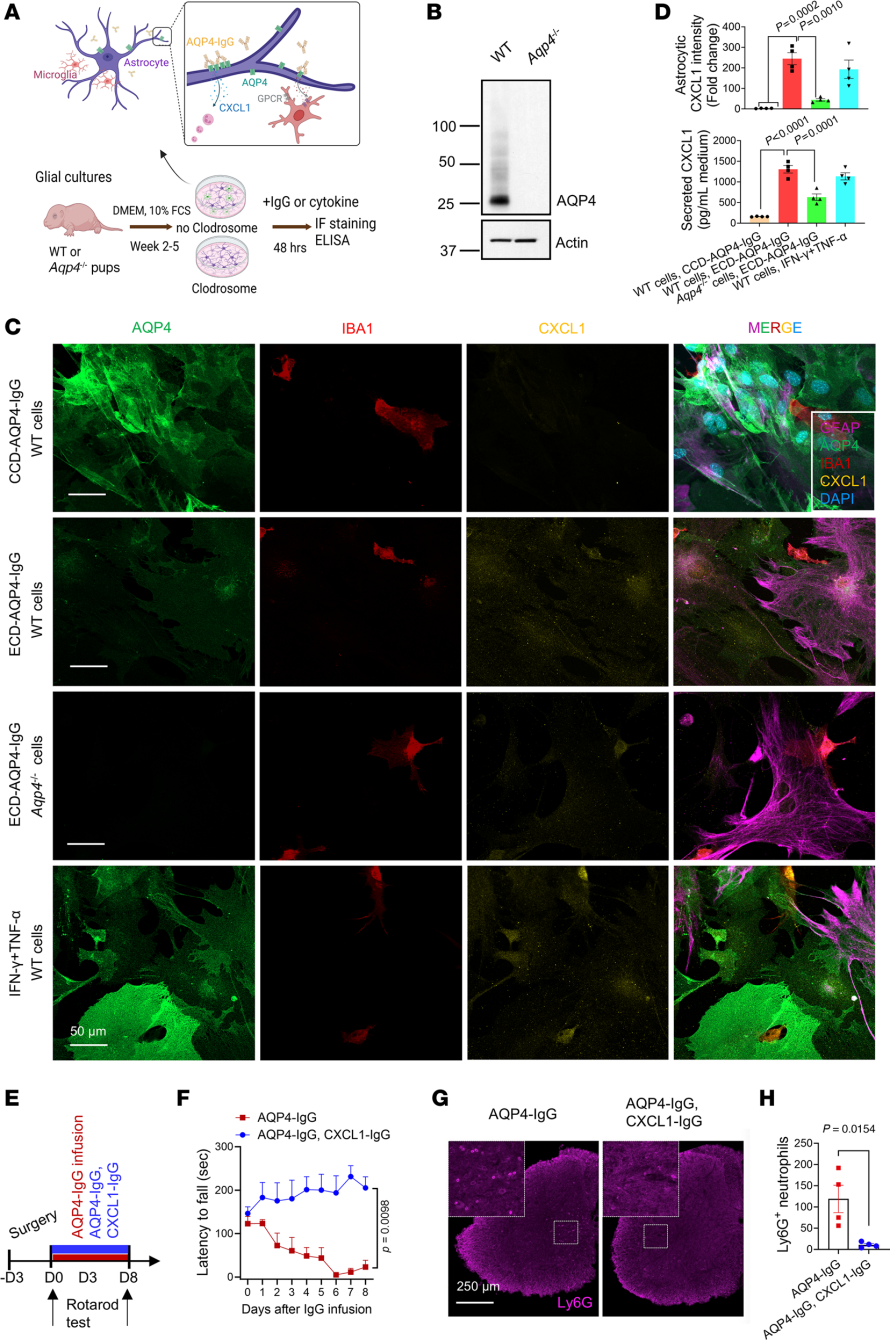
Microglia are required for pathogenic AQP4-IgG to upregulate CXCL1 in cultured mouse astrocytes. (**A**) Experimental design of the IgG binding to AQP4 on astrocytes in primary glial cultures established from wild-type and *Aqp4^–/–^* pup brains. Microglia were depleted by treating Clodrosome (100 μg/mL) before subculture. Two days after adding IgG or cytokines, CXCL1 production was assessed by immunostaining or ELISA. (**B**) Immunoblot analysis of WT, *Aqp4^–/–^* primary glial cells using IgGs specific for AQP4 and actin (loading control). (**C**) CXCL1 immunoreactivity was assessed in wild-type and *AQP4*± cells exposed to a control nonpathogenic monoclonal mouse IgG specific for the AQP4 cytoplasmic C-terminal domain (CCD-AQP4-IgG, m5) or a pathogenic extracellular domain–reactive IgG (ECD-AQP4-IgG, m21) or to IFN-γ plus TNF-α cytokines. Astrocytes are identified by AQP4 and GFAP immunoreactivities; microglia by IBA1; DNA is blue (DAPI). (**D**) Cellular CXCL1 (upper) was quantified from fluorescence intensity in **C** images; secreted CXCL1 protein levels (lower) were quantified in the glial culture media by ELISA. (**E**) Experimental design: 2 groups of WT mice were infused continuously via spinal subarachnoid catheter (day 0 to day 7) with pathogenic ECD-AQP4-IgG or ECD-AQP4-IgG mixed with CXCL1-IgG. (**F**) Motor function of mice in **E** was assessed by rotarod test; time: *F*_(8,__40)_ = 1.319, *P* = 0.2632; treatment: *F*_(1,__5)_ = 16.38, *P* = 0.0098; interaction: *F*_(8,__22)_ = 7.766, *P* < 0.0001; *n* = 4–5 mice per group. (**G** and **H**) Representative images at day 7 of IgG infusion and quantification of neutrophils (Ly6G^+^) in lumbar cord parenchyma (*n* = 4 mice per group). Higher magnifications (left corners) of boxed areas in **G** show neutrophils (magenta). One-way ANOVA with Tukey’s post hoc in **D**; *n* = 4 wells in **D**; 2-way repeated measures ANOVA with Holm-Šídák post hoc test in **F**; *t* test in **H**.

Early experimental models of NMO, both in vitro (26) and in vivo (35), implicated a pathogenic role for granulocytic CNS infiltration. Astrocyte-derived CXCL1 is known to drive neutrophil transmigration in mouse models of viral encephalitis (36) and ischemic stroke (37). To investigate whether CXCL1 protein production is upregulated following ligation and internalization of astrocytic AQP4 by IgG, we incubated WT or *Aqp4*^–/–^ mouse astrocytes with pathogenic or nonpathogenic monoclonal AQP4-IgGs; IFN-γ plus TNF-α served as a control activator of astrocytes (Figure 7A). *Aqp4*^–/–^ mouse astrocytes were confirmed AQP4 deficient by immunoblotting (Figure 7B). Compared with the effect of nonpathogenic C-terminal cytoplasmic domain–reactive AQP4-IgG (mCCD, control), the immunofluorescence staining intensity of cytoplasmic CXCL1 was increased 244-fold (*P* = 0.0002; Figure 7, C and D) after 48 hours’ exposure to the pathogenic AQP4-IgG (mECD, extracellular domain–reactive), and secreted CXCL1 levels (detected by ELISA) rose significantly (1,305 pg/mL versus 159 pg/mL; *P* = 0.0010; Figure 7, C and D). However, in WT glial cultures depleted of microglia, pathogenic AQP4-IgG increased CXCL1 production only 8-fold (Supplemental Figure 10). Furthermore, the cytoplasmic CXCL1 increase was significantly attenuated when pathogenic AQP4-IgG was applied to *Aqp4*^–/–^ astrocytes, despite co-culture with microglia (Figure 7D; upper, *P* = 0.0010; lower, *P* = 0.0001). These findings indicate that upregulated astrocytic production and secretion of CXCL1 by AQP4-IgG requires microglia-astrocyte interaction.

To investigate whether blockade of CXCL1-mediated granulocytic trafficking in vivo might attenuate motor dysfunction in this mouse NMO model, we co-infused anti-CXCL1-IgG together with AQP4-IgG or control-IgG into the subarachnoid space for 7 days (Figure 7E). Behavioral testing revealed that inhibition of CXCL1 signaling prevented motor impairment by AQP4-IgG infusion. The motor performance of mice treated with anti-CXCL1-IgG (Figure 7F and Supplemental Video 5) was comparable to that of neutrophil-depleted mice (Figure 3G). Moreover, CXCL1 signaling blockade prevented neutrophil infiltration into the spinal cord in this NMO model (Figure 7, G and H). Thus, CXCL1-dependent neutrophil recruitment is a critical step in initiating an NMO attack.

### A subpopulation of disease-associated microglia (dual positive for Galectin-3 and P2Y12) interacts with motor neurons.

**Figure 8:**
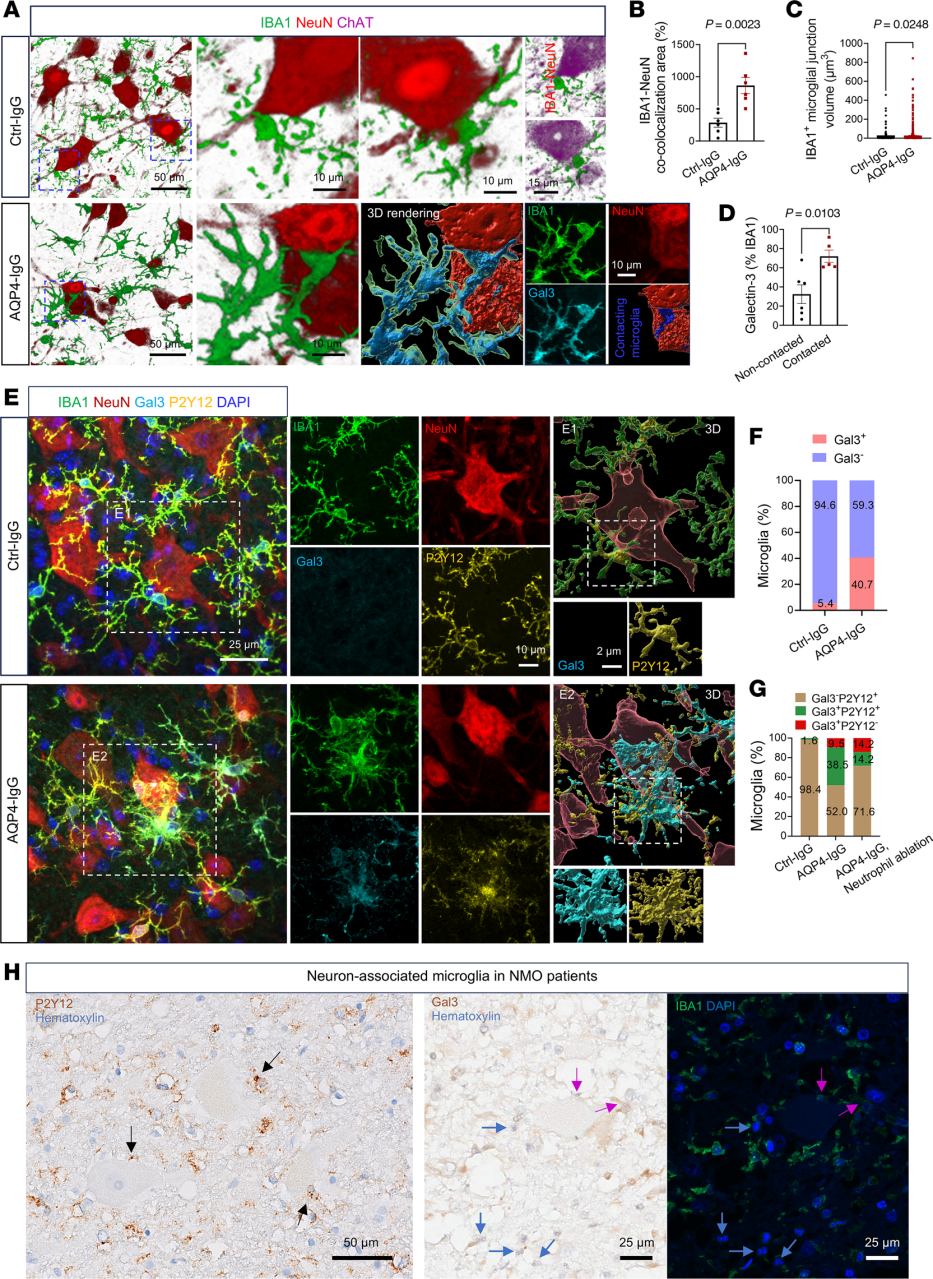
Disease-associated microglia interact with motor neurons in an NMO mouse model. (**A**) Representative immunostained images of motor neuron–associated (ChAT^+^) microglia (IBA1^+^) in ventral gray matter of mice infused for 3 days with IgG infusion. Boxed areas are enlarged on the right. 3D rendering of boxed area in AQP4-IgG mice is shown as split confocal channels (lower right) and merged (lower center). (**B** and **C**) Quantification of microglia-neuron co-colocalization area (**B**) and volume (**C**) in **A** (*n* = 6 mice per group in **B**; *n* = 1,526 and 1,272 microglial contacts, respectively, for Ctrl-IgG and AQP4-IgG mice). (**D**) ImageJ analysis shows the percentage area occupied by Galectin-3 within contacting microglia is greater than in noncontacting microglia in lumbar cord of the 2 experimental groups. (**E**) Overlaid confocal images show microglial P2Y12 receptor (yellow), Galectin-3 marker of DAMs (cyan) contacting neurons in lumbar ventral gray matter of AQP4-IgG-infused mice, in contrast with control-IgG–infused mice. (**F**) Percentage of DAMs (IBA1^+^Gal3^+^) contacting neurons is significantly increased after AQP4-IgG infusion. (**G**) The enhanced expression of P2Y12 receptor by IBA1^+^Gal3^+^ DAMs (38.5%) contacting neurons implicates P2Y12 in the contact mechanism. (**H**) Case 1 (left) immunohistochemistry reveals microglia (P2Y12^+^, brown) associated with neurons in this early stage in spinal cord ventral gray matter lesion of an NMO patient. Arrows identify microglia-neuron contact sites. Case 2 (middle and right) immunohistochemistry combined with immunofluorescence reveals Galectin-3 (brown; IHC) expressed in neuron-associated IBA1^+^ microglia (green; IF) in an early lesion of a second NMO patient’s spinal cord ventral gray matter. Magenta arrows identify microglia (Galectin-3^+^IBA1^+^) associated with motor neurons; blue arrows identify infiltrated neutrophils in this area. Statistics in **B**–**D** used *t* test.

To investigate how microglia-neuron interactions might modulate neuronal function, we analyzed the surface phenotype of microglia in the ventral horn of mice by immunostaining microglial IBA1 and motor neuronal ChAT after infusing AQP4-IgG for 72 hours. 2D and 3D analyses revealed a significant increase in microglial contacts with somata of motor neurons in AQP4-IgG recipients (Figure 8, A–C) in comparison with control-IgG–infused mice. In AQP4-IgG recipients, the signal area for Galectin-3, a marker of disease-associated microglia (DAM), was 122% greater in microglia contacting neurons compared with microglia not contacting neurons (Figure 8D). Microglia are known to regulate neuronal activity by sensing neuron-derived ATP/ADP via the P2Y12 purinergic receptors enriched on their processes (31, 38–40). In the context of “NMO” disease evolution in AQP4-IgG–recipient mice, we found high expression of P2Y12 receptors on 38.5% of Galectin-3^+^ microglial clusters interacting with motor neurons (Figure 8, E and F). Further, these “NMO” DAM clusters were substantially fewer in the spinal cords of neutrophil-ablated mice (Figure 8G). Immunohistochemical analysis of a sublytic pre-demyelinated lesion in an archival NMO patient’s spinal cord that had lost AQP4 revealed P2Y12^+^ microglia (Figure 2, G–J, and Figure 8H, NMO case 1). A nondestructive demyelinated lesion from another patient with NMO contained Galectin-3^+^IBA1^+^ microglia and neutrophils (Figure 8H, NMO case 2) close to motor neurons. We interpret the unprecedented finding of dual expression of a disease-associated marker (Galectin-3) and a homeostatic marker (P2Y12) on motor neuron–associated microglia in the early precytolytic ventral spinal cord lesions of our mouse NMO model as evidence of a potentially previously unrecognized, transitional-state microglial subpopulation responding to infiltrating neutrophils.

## Discussion

Neutrophil infiltration is a histopathological feature that distinguishes NMO from other inflammatory CNS demyelinating disorders (5). Our findings in a mouse model of NMO assign neutrophils a critical, therapeutically targetable role in initiating motor neuronal dysfunction prior to activation of the classical complement cascade and ensuing irreversible tissue destruction. Analogous to observations in early CNS lesions of patients with NMO (8), spinal subarachnoid infusion of a pathogenic, non-complement-activating monoclonal AQP4-IgG into mice causes neutrophil migration into the cord parenchyma to undergo NETosis. Our initially reported mouse NMO model identified a C3a/C3aR1 signaling–dependent interaction between AQP4-IgG–activated astrocytes and microglia as a driver of initial lesion formation and motor impairment (16). Our present study demonstrates that neutrophils are recruited soon after in vivo engagement of pathogenic IgG with astrocytic AQP4, with neutrophil-microglia interaction via C5a/C5a receptor signaling being a critical co-stimulatory event for motor paresis development. Surprisingly, we found no C5 protein in the mouse NMO model’s evolving CNS lesion, but C5a immunoreactivity was abundant in the cytoplasm of CNS-infiltrating neutrophils and in peripheral neutrophils activated in vitro. We concluded that C5 was synthesized by activated neutrophils, rapidly cleaved posttranslationally by cytoplasmic cathepsins, and secreted as C5a (41, 42). Thus, our study suggests the complosome of CNS-infiltrating neutrophils is a noncanonical source of C5a synergizing with astrocyte-derived C3a (16) to co-stimulate microglia and serving as a cell-autonomous mechanism enhancing neutrophil influx.

In the context of aging and neurodegenerative diseases, neuronal oxidative stress induces toxic lipid droplet accumulation in astrocytes (43). This is attributed to impaired transfer of lipid products from neuron to astrocyte for mitochondrial catabolism by β-oxidation (44). Immunohistopathological studies of sublytic astrocytopathy in CNS tissues of NMO suggest that damage signals from lesional astrocytes are propagated via the panglial syncytium to distant astrocytes that are not themselves ligated by IgG (i.e., they retain surface AQP4) (45). It is conceivable that microglia, fully activated by astrocytic C3a (16) and neutrophilic C5a (present data), are primed to release factors that further promote astrocytopathy, such as by impairing the processing of lipids transferred from neurons. Important observations in our present study are 1) neurochemical evidence of motor neuronal oxidative stress, loss of ChAT, and lipid droplet accumulation accompanying initial clinical motor impairment that is reversible on removal of pathogenic AQP4-IgG and 2) motor dysfunction preceding endothelial tight junction disruption. We therefore propose that the hallmark complement-mediated cytodestructive end-stage lesions responsible for irreversible NMO disability require gross BBB disruption to allow astrocytic membrane lesioning by deposition of plasma-derived opsonic macromolecules, including IgM and C1q (5). It is at this point that neutralization of circulating and CNS-penetrating C5 proenzyme by anti-C5 monoclonal antibody therapy (eculizumab) is highly efficacious in aborting an acute attack and preventing NMO relapse (46). Our study demonstrates that C5a/C5aR1 signaling between infiltrating neutrophils and resident microglia (the predominant C5aR1-expressing cells in the CNS) is an early pathogenic mechanism contributing to neuronal stress and motor dysfunction.

We discovered several cellular mechanisms that plausibly underlie both the evolving histopathological lesion and motor impairment. Neutrophil-attractant chemokines (CXCL1, CXCL5, CXCL7, CXCL8) and activation markers (MPO and NE) are elevated in attack-phase CSF of patients with NMO (47, 48). Our glial culture experiment demonstrated that AQP4-IgG stimulates astrocytic production and release of CXCL1. The far lower CXCL1 levels induced by IgG in *Aqp4*^–/–^ astrocytes and in microglia-depleted cultures indicate this mechanism is both antigen dependent and microglia dependent. It is yet to be determined whether CNS infiltration by neutrophils is solely dependent on CXCL1 secretion by activated astrocytes.

Recognition by clinicians that neutrophils and their chemokines aid the distinction of NMO from multiple sclerosis (7), and that neutrophil infiltration correlates with lesion size in patients with NMO (8), underscores the therapeutic relevance of our findings. The demonstration by Saadoun et al. (9) that administration of neutrophil protease inhibitors limited the lesion size in mice with disrupted BBB (injected intracerebrally with NMO patient IgG combined with fresh human complement) highlighted the significance of neutrophils as therapeutic targets in the cytodestructive phase of NMO. Our study has revealed a critical earlier role for neutrophils in initiating NMO pathophysiology prior to tissue damage.

Targeting of neutrophils or C5a receptors is an attractive therapeutic option for attack intervention in NMO. Avacopan, a C5aR1 antagonist, is FDA approved for treating anti-neutrophil cytoplasmic antibody–associated vasculitis (49). The BBB-permeable C5aR1 inhibitor, PMX 53, was demonstrated to prevent astrocytic lysis in rats co-injected intracerebrally with AQP4-IgG and human serum as an exogenous source of complement components (35). Our study highlights the critical pathophysiological importance of neutrophil-microglia interaction preceding activation of the classical complement cascade and resultant cytodestruction, by demonstrating that motor paresis can be mitigated by the C5aR1 antagonist, PMX 205, in the precytolytic phase of NMO. The release of proteolytic enzymes, bioactive lipids, and reactive oxygen species by degranulating neutrophils is considered to cause neuronal oxidative stress and may indeed contribute to motor neuron dysfunction in the progressing NMO lesion (50–52), but those events occur later than the time frame of neurological impairment addressed in our study. Activated astrocytes (36) and secondarily activated neural, endothelial, T, and myeloid cells, both resident and infiltrating, all undoubtedly contribute to NMO pathology (53). However, the molecular contributions of microglia to oxidative stress and lipid droplet accumulation in motor neurons remain poorly understood, as does the basis for selective vulnerability of motor neurons. Our identification of a Galectin-3^+^ subpopulation of DAM-contacting motor neurons provides a direction for future investigations (54, 55). This microglial subpopulation, identified at day 3 of AQP4-IgG infusion in the evolving spinal cord lesion of our mouse NMO model, represents an “intermediate” or “transitioning” state between the P2Y12^+^ homeostatic phenotype and the Galectin-3^+^ disease-associated phenotype of activated microglia. The NMO patients’ spinal cord specimens available for our study may have missed this temporal window of microglial phenotypic transition due to differing stages of evolution in multifocal lesions, the influence of antiinflammatory therapies, or intrinsic immunobiological differences between humans and mice. We hypothesize that Galectin-3 protein observed on microglia activated by infiltrating neutrophils and contacting motor neurons in early NMO patient lesions (P2Y12/Galectin-3 double positive in the NMO mouse) have a functional role in cell contact and in NMO lesion evolution. Spatial and single-cell proteomics should further elucidate the complex neutrophil–microglial interactions driving early NMO pathogenesis.

## Methods

### Sex as a biological variable.

This study included only female mice, reflecting the strong female predominance observed in NMO, with reported female-to-male ratios of up to 9:1 (56, 57).

### Mice.

We used 8- to 12-week-old female C57BL/6J WT mice, *Cx3cr1*^GFP/+^ transgenic mice, and *C5ar1*^–/–^ mice. C57BL/6J (stock no. 000664), *Cx3cr1*^GFP/GFP^ [B6.129P2(Cg)-*Cx3cr1*^tm1Litt^/J, stock no. 005582], *C5ar1*^–/–^ [B6.129S4(C)-*C5ar1*^tm1Cge^/BaoluJ, stock no. 033903], and *CCR2*^CreER-GFP^ [C57BL/6-*Ccr2*^em1(icre/ERT2)Peng^/J, stock no. 035229] mouse lines were purchased from The Jackson Laboratory and bred at Mayo Clinic. Mice were group-housed on a 12-hour light/dark cycle in a temperature- and humidity-controlled room. *Cx3cr1*^GFP/+^ mice were used to visualize microglia and neutrophil interaction for electron microscopy imaging.

### Spinal catheter placement and IgG delivery.

Under isoflurane anesthesia, we inserted into the subarachnoid space via the cisterna magna (through a 1 mm horizontal incision in the dura mater) a 6 cm polyurethane intrathecal catheter (ALZET, no.: 0007743), facilitated by a Teflon-coated stainless steel stylet, and extended it to the lumbar spinal level. Dental glue was applied to prevent leaks from catheter connections, and the catheter was anchored by suturing to neck musculature. On awakening, mice were observed postoperatively for 2 hours for motor signs of traumatic cord injury. Injured mice (~10%) were promptly euthanized. After 3 days’ training on the rotarod beam (days –3 to –1, 4 rpm/min, 5 min) with daily slow manual catheter flushing with artificial CSF, an osmotic minipump delivery system (ALZET, 1007D), containing a pathogenic mouse monoclonal anti-AQP4-IgG1 (clone mECD, isotype, IgG1κ, AQP4-m21, created in-house; licensed to Sigma-Aldrich as MABN2471) or mouse polyclonal serum IgG (Sigma, I5381), or anti-AQP4-IgG plus rat monoclonal anti-CXCL1-IgG (R&D Systems, MAB453, clone 48415, 0.5 μg/12 μL/day for 8 days), was placed subcutaneously between the shoulders. AQP4-IgG or mouse serum IgG was infused continuously for 1, 3 or 7 days (1.2 μg/12 μL/day). clone mECD induces clustering and internalization of AQP4 and causes its redistribution, as is also observed with NMO patients’ IgG. (58).

### Rotarod training and testing.

Daily testing began day 0, at 4 rpm/min and accelerated to 40 rpm/min over a 5-minute period. Each mouse underwent 3 trials at 15-minute intervals. The recorded latency to fall from the rotarod beam was averaged for each mouse.

### Tissue collection and immunofluorescence staining.

Spinal cord tissues were harvested following euthanasia and transcardiac perfusion with 30 mL ice-cold PBS. After 10% formalin perfusion, fixation (16–24 hours at 4°C), then cryoprotection by immersion in 30% sucrose, tissues were cryosectioned coronally (16–40 μm thick, LEICA cryostat CM1520) and embedded in Tissue-Tek OCT compound (Fisher HealthCare) (Figures 1 and 4); 50 μm thick sections were prepared by vibratome (LEICA VT1000S) without embedding (all other figures) for immunofluorescence staining. We mounted 16 μm sections directly onto SuperFrost Plus microscope slides (Fisher Scientific) (stored at –80°C) and prepared 40 μm and 50 μm sections for free-floating staining (stored in 30% glycol). For immunofluorescence staining, sections were permeabilized and blocked (30 min), using 0.25% Triton X-100 in PBS containing 2.5% bovine serum albumin and 5% donkey serum, then exposed 16–24 hours at 4°C to the following primary antibodies in PBS containing 0.1% Triton X-100 (Sigma) and 1% BSA: rat anti-Ly6G (Bio X Cell; BE0075-1, clone 1A8, 1:500), human/mouse polyclonal goat anti-MPO (R&D Systems; AF3667; 1:800), rat anti-NE (R&D Systems, MAB4517-SP, clone 887105, 1:500), mouse anti-C5 (Hycult Biotech; HM1073, clone BB5.1, 1:500), rat anti-C5a (Invitrogen; MA5-23910, clone 295108, 1:300), goat polyclonal anti-GFP (Abcam; Ab6673, 1:1,000), polyclonal goat anti-CD31 (R&D Systems; AF3628, 1:500), rabbit antiIBA1 (Abcam; Ab178846, clone EPR16588, 1:500), polyclonal goat antiIBA1 (Wako; 011-27991, 1:500), guinea pig antiIBA1 (Synaptic Systems; 234 308, clone Gp311H9, 1:1,000), rabbit polyclonal anti-ChAT (Sigma-Aldrich, AB143; 1:100), rabbit polyclonal anti-P2Y12 (AnaSpec; AS-55043A; 1:1,000), human anti-HuD (59) (from patients with paraneoplastic neurological autoimmunity related to small-cell lung carcinoma; 1:10,000), polyclonal goat anti-human/mouse/rat Galectin-3 antibody (R&D Systems; AF1197, NP_034835, 1: 1,000), and mouse anti-4-HNE (R&D Systems; MAB3249-SP, clone 198960, 1:200). Sections were then exposed (22°C, 2 hours) to appropriate fluorochrome-conjugated secondary antibodies (diluted in PBS containing 0.1% Triton X-100 and 1% BSA) and then counterstained with DAPI (Sigma-Aldrich, 0.1 μg/mL) and mounted with SlowFade Diamond Mountant (Thermo Fisher Scientific). Images were acquired using either a ×63 oil-immersion objective lens or a ×40 water-immersion objective lens with a ZEISS LSM980 confocal microscope, ensuring optimal resolution for different experimental setups. For EM imaging of neutrophil-microglia interaction, the cord was expelled from the vertebral canal after PBS perfusion using ice-cold PBS from a 3 mL syringe connected to a 200 μL pipette. Lumbar cord (2 mm length) was quickly immersed (2 hours at 4°C) in 4% EM-grade paraformaldehyde aqueous solution (Electron Microscopy Sciences, no. 15710, diluted 1:4 in PBS). Vibratome sections were prepared without embedding (50–100 μm, LEICA VT1000S). The immunostaining method was standard except that: 1) primary antibodies (Bio X Cell; BE0075-1, rat anti-Ly6G, 1:200, and Abcam; Ab104225, rabbit polyclonal anti-NeuN, 1:500), and Alexa Fluor–conjugated secondary antibodies (donkey anti-rat IgG, 594, [A-21209, Thermo Fisher Scientific], and donkey anti-rabbit IgG, 405; [ab175651, Abcam] were diluted 1:500) in PBS without permeabilization; 2) secondary antibody exposure was at 4°C for 24 hours, with mild shaking; and 3) cord sections were mounted in 30% glycerol on glass slides for confocal imaging. For EM imaging of lipid droplets, lumbar cord (2 mm length) was quickly dissected and postfixed in 2% glutaraldehyde and 2% paraformaldehyde in 0.1 M cacodylate buffer containing 2 mM calcium chloride without immunostaining. For immunofluorescence staining of autopsied FFPE human tissues, TrueBlack lipofuscin autofluorescence quencher (Biotium, catalog 23011) diluted in 70% EtOH was applied after immunofluorescence staining before mounting in SlowFade Diamond Mountant. Surface rendering, spot rendering, filament rendering, and visualization were performed using Imaris v.10.0 (Oxford Instruments).

### Immunohistochemical analysis of human spinal cord.

An archival block of spinal cord from an autopsied NMO patient was subjected to neuropathological evaluation, as previously detailed (45). Sections (5 μm) of paraffin-embedded, formalin-fixed tissue were stained with hematoxylin-eosin or Luxol fast blue/periodic acid–Schiff myelin stain. Immunohistochemistry utilized an EnVision FLEX + secondary system (Dako, Agilent) (60). Primary IgG antibodies included polyclonal rabbit anti-AQP4 (Sigma; A5971; 1:250), KiM1P (monoclonal, gift from Wolfgang Brück, University Medical Center Göttingen, Göttingen, Germany), rabbit anti-human P2Y12 (Abcam; Ab300140, clone EPR26298-93, 1:1,000), and polyclonal goat anti-human/mouse/rat Galectin-3 antibody (R&D Systems; AF1197, NP_034835, 1:100). Images (×40) were captured using an Olympus BX51 microscope (EVI-DENT).

### Single-cell suspension for flow cytometry.

Spinal cords from 3 cohorts of mice were perfused and harvested after 1, 3, and 5 days’ subarachnoid infusion with AQP4-IgG; cord tissue from a control cohort infused with normal mouse serum IgG was harvested on day 3. All mice were terminally perfused with ice-cold PBS. Lumbar cords were rapidly harvested, digested in HBSS containing collagenase I/IV and DNase, and shaken (New Brunswick Scientific NB-C24; 37°C, 1*g*, 35 min), with trituration by polished glass pipette every 10 min to aid dissociation (61). After passage through 70 μm nylon mesh (Miltenyi Biotec, no. 130-110-916), the suspension was centrifuged (515*g*, 7 min at 4°C), and the pellet was resuspended in PBS, gently overlaid on 30% Percoll, and centrifuged (800*g*, 20 min at 4°C). Sedimented cells, free of debris and myelin, were resuspended in ice-cold PBS, counted, filtered via 70 μM cell strainer (Falcon no. 352052), immunostained, and phenotyped by flow cytometry.

### High-parameter flow cytometry and analysis.

A preliminary experiment determined optimal antibody dilutions and appropriately set compensation for the antibody panel’s multiple fluorochromes (62). After resuspension in PBS, cells were first stained with Zombie UV viability dyes (BioLegend, 1:1,000; 20 min at room temperature in the dark). Fc-receptors were blocked using rat IgG2b anti-mouse CD16/CD32 (BioLegend, 101302, clone 93, 1:50), and the following mixed panel of surface-staining antibodies was applied (30 min, 4°C): CD45-PE-CF594 (BD Biosciences, 562420, clone 30-F11, 1:1,000), CD11b-PE-Cy5 (Tonbo, 55-0112-U100, clone M1/70, 1:1,000), CD11c (BioLegend, 117334, clone n418, 1:200), CD3-PE/Fire700 (BioLegend, 100272, clone 17A2, 1:200), CD4-BV510 (BioLegend, 100449, clone GK1.5, 1:200), CD8a-BV785 (BioLegend, 100750, clone 53-6.7, 1:200), CX3CR1-Pacific Blue (BioLegend, 149038, clone SA011F11, 1:300), CD206-BV711 (BioLegend, 141727, clone C068C2, 1:200), Ly6G-APC-Cy7 (BioLegend, 127623, clone 1A8, 1:200), Ly6C-PerCP (BioLegend, 128028, clone HK1.4, 1:100), TMEM119-PE-Cy7 (eBioscience, 25-6119-82, clone V3RT1Gosz, 1:200), C3aR-BV480 (BD Biosciences; 753370, clone 14D4, 1:200), C5aR-PE (BioLegend, 135805, clone 20/70, 1:100), NK1.1-APC/Fire 810 (BioLegend, 156519, clone S17016D, 1:200), MHC-II-BB700 (BD Biosciences, 746197, clone M5/114.15.2, 1:300), P2ry12-APC (BioLegend, 848005, clone S16007D, 1:500), and B220-Spark NIR685 (BioLegend, 103268, clone RA3-6B2, 1:200). After washing, the samples’ emission spectra were measured on a 5-laser Aurora cytometer (Cytek; Spectro Flo software). The data were analyzed using FlowJo v10.10 Software (Waters Biociences).

### Near-infrared branding.

We acquired whole-mount confocal images of sections with ×20 lens, tiles, and *Z*-stack, then enlarged (×63 oil lens with *Z*-stack) foci of interest containing neutrophils adjacent to microglia (Ly6G/Cx3cr1-GFP). The spinal cord section was then rinsed and plated in 1× PBS on a new glass microscope slide. We generated fiducial branding marks on spinal cord sections by progressively increasing the laser power of a 2-photon microscope at micrometer-precision points. The holes created by induced microbubbles enabled later reidentification of the field containing microglia and neutrophils of interest. The section was next postfixed (4°C, 24 hours) in 2% glutaraldehyde and 2% paraformaldehyde in 0.1 M cacodylate buffer containing 2 mM calcium chloride.

### SBF-SEM.

SBF-SEM was performed as previously described (63). The workflow combined the 2 techniques to allow detection of individual regions of interest in a large field at small X, Y pixel size and imaging of the subsequent targeted volume at high voxel resolution. EM imaging was acquired under high-vacuum/low-water conditions with a starting energy of 1.8 keV and beam current of 0.10 nA. Stacks of several hundred sections (100 nm thick, obtained by the diamond knife) were imaged at 15 nm × 15 nm × 100 nm spatial resolution. By aligning with *Z*-stack confocal images, the targeted microglia-neutrophil couple can be reconstructed based on the automatically generated EM-level 3D database. For analysis, Fiji-ImageJ (NIH) and Reconstruct software packages were used to identify cell morphology, interaction, ultrastructure, and segmentation.

### Pharmacological inhibition of C5aR1 signaling in vivo.

Trifluoroacetate was removed from the cyclic hexapeptide C5aR1 antagonist PMX 205 (HY-110136 A; MedChemExpress) by repeated hydrochloric acid exchange followed by lyophilization (64). For pharmacological inhibition of C5aR1 signaling, we injected the purified peptide (10 mg/kg) subcutaneously 4 times on alternate days after commencing AQP4-IgG infusion. PBS was injected into a sham-treated group.

### Lipid droplet staining and analyses.

Lipid droplet (LD) staining was slightly modified from a previous description (44). Postfixed lumbar cord sections were rinsed in 1× PBS for 5 min and incubated in 1× PBS containing 0.25% Triton X-100 before 10-minute incubation with BODIPY 493/503 (Thermo Fisher Scientific, D3922, 1 μg/mL). To prevent dye washout, sections were transferred directly from the staining solution to glass slides without additional washing. To image LDs, we used a ZEISS LSM980 confocal microscope at ×63 magnification (2,048 × 2,048 pixels, 0.5 μm *Z*-steps), keeping constant the *Z*-slice numbers per lumbar section within each experiment. For unbiased quantification of LD numbers, we used the “Spot model” for LD counts and the “Surface model” for representative images in Imaris v10.0.

### Mouse astrocyte culture and immunostaining.

*Aqp4*^–/–^ mice were created and bred in-house (Mayo Clinic). Mixed glial cultures were established from newborn mouse cerebral cortices as previously reported (58) and used within 5 weeks. Microglia were depleted by adding Clodrosome (100 μg/mL; Encapsula Nano Sciences LLC) to growth medium (DMEM, 10% FCS, sodium pyruvate, glutamine, antibiotics) for 72 hours at 2–5 weeks after initial plating and depletion was confirmed by immunostaining with guinea pig antiIBA1 IgG (Synaptic Systems; No. 234 308, clone Gp311H9, 1:1,000). Primary glia cultures were treated with 300 ng/mL LPS (Sigma, L2880) and 300 U/mL IFN-γ (Gibco, 315-05) plus 25 ng/mL TNF-α (Gibco, 315-01A) diluted in Gibco DMEM (11965-084) and 1% penicillin/streptomycin for 48 hours. Glial cell culture supernatant was collected and stored at –80°C for mouse KC/CXCL1 ELISA (Sigma, RAB0117). The ELISA was conducted in accordance with the manufacturer’s instructions (Sigma-Aldrich). For immunofluorescence experiments, astrocytes were plated on glass coverslips with a uniform application of poly-d-lysine and laminin (Corning BioCoat; 354087), postfixed in 4% PFA for 15 min, washed, permeabilized 5 min in PBS/0.02% Triton X-100, and blocked in 3% donkey serum, 30 min. Cells were incubated in primary antibodies at 4°C for 16 hours, washed in 1× PBS, and exposed to secondary antibodies, mounted with SlowFade Diamond Antifade Mountant (Thermo Fisher Scientific), and imaged 1 plane by ZEISS 980 microscope. Primary antibodies were mouse anti-GFAP (Abcam; Ab4648, clone 2A5, 1:1,000), polyclonal rabbit anti-AQP4 (Sigma; A5971, 1:500), guinea pig antiIBA1 (Synaptic Systems, 234 308, clone Gp311H9, 1:1,000), and rat anti-CXCL1 (R&D Systems, MAB453, clone 48415, 1:200). Alexa Fluor–conjugated secondary antibodies (donkey anti-mouse IgG, 647, donkey anti-rabbit IgG, 488, donkey anti-guinea pig Cy3, and donkey anti-rat IgG, 594) were diluted 1:500 in PBS without permeabilization for 2 hours at room temperature. Data represent neurons (*n*) from multiple independent experiments.

### Neutrophil and microglia ablation.

To deplete peripheral neutrophils, mice were injected twice i.p. with anti-mouse Ly6G (*InVivo*MAb, Bio X Cell, BE0075-1, clone: 1A8, 100 mg/kg) or isotype control rat IgG2a, at 5 and 3 days before rotarod testing. The following antibodies identified mature neutrophils (Gr1^hi^CD11b^+^, Gr^hi^MPO^+^) (65), immature myeloid cells (Gr1^int^CD11b^+^) (65), and immature neutrophils or granulocytic precursors (Gr1^int^MPO^+^) (66, 67) in peripheral blood using spectral flow cytometry (Cytek Aurora, Cytek Biosciences): CD45-violetFluor 450 (Tonbo, 75-0451-U100, clone 30-F11, 1:1,000), CD45-PE-CF594 (BD Biosciences, 562420, clone 30-F11, 1:1,000), CD11b-PE-Cy5 (Tonbo, 55-0112-U100, clone M1/70, 1:1,000), Ly6G-APC-Cy7 (BioLegend, 127623, clone 1A8, 1:200), Ly6C-PerCP (BioLegend, 128028, clone HK1.4, 1:100), and Gr1-PE (BioLegend, 108408, clone RB6-8C5, 1:200). After exposure to surface antibody panel, cells were washed, permeabilized by Cytofix/Cytoperm solution (BD Biosciences 554722) at 4°C for 20 min, then exposed sequentially to polyclonal goat anti-MPO IgG (R&D Systems, AF3667, 1:1,000; 30 min at 4°C), then donkey anti-goat IgG (Alexa Fluor 488–conjugated, Invitrogen, A32814TR; 30 min at 4°C); both antibodies were diluted in 1× Perm/Wash buffer (BD Biosciences 554723). To ablate microglia, mice were fed ad libitum chow containing PLX3397 (CSF1R inhibitor, 600 mg/kg, Chemgood, C-1271) for at least 3 weeks prior to surgery and behavioral tests.

### Statistics.

All statistical analyses were conducted by GraphPad Prism v8 (GraphPad Software Inc.). Normality and variance were tested using the Shapiro-Wilk test. For comparisons between 2 groups, an unpaired 2-tailed Student’s *t* test was performed. When comparing 3 groups, 1-way ANOVA followed by Tukey’s post hoc test was used. For comparisons involving 4 groups with 2 independent variables (e.g., treatment × genotype), 2-way repeated measures ANOVA with Holm-Šídák post hoc test were applied (e.g., in experiments validating *C5ar1* deficiency). Additionally, for repeated measures, 2-way repeated-measures ANOVA with Holm-Šídák post hoc test was used, as in the rotarod test. Sample sizes were determined based on previous studies utilizing similar methodologies. Data are presented as mean ± SEM. A *P* < 0.05 was considered statistically significant; nonsignificant results (*P* > 0.05) are not displayed.

### Study approval.

Animal procedures and protocols were approved by the Mayo Clinic Institutional Animal Care and Use Committee (protocol no. 2909-17) and were conducted in agreement with the NIH *Guide for the Care and Use of Laboratory Animals* (National Academies Press, 2011) Use of consented autopsy tissues was approved by Mayo Clinic Institutional Review Board (IRB#2067-99).

### Data availability.

All data supporting the findings of this study are provided in the article, its supplement, and its Supporting Data Values file or upon reasonable request. Further information can be found in Supplemental Methods.

## Author contributions

FQ, LJW, and VAL designed research studies. SZ, TJC, and YG advised on methodology related to surgery models and laser branding under 2-photon imaging. HD conducted and acquired experiments related to Western blot. YG performed immunohistochemistry on human samples. CL performed rotarod behavioral test related to C5aR1 antagonist. WMB performed glial cell cultures. FQ prepared the original manuscript. FQ, LJW, CFL, and VAL reviewed and edited the manuscript and provided important scientific advice.

## Funding support

This work is the result of NIH funding, in whole or in part, and is subject to the NIH Public Access Policy. Through acceptance of this federal funding, the NIH has been given a right to make the work publicly available in PubMed Central.

- NIH grants R01 NS110949 (LJW and VAL) and R35 NS132326 (LJW).

## Supplementary Material

Supplemental data

Unedited blot and gel images

Supplemental video 1

Supplemental video 2

Supplemental video 3

Supplemental video 4

Supplemental video 5

Supporting data values

## Version 1 02/10/2026

In-Press Preview

## Version 2 04/01/2026

Electronic publication

## Version 3 04/07/2026

correction to an author affiliation
