# Supplementary material for: Neutrophil-microglia interaction drives motor dysfunction in a neuromyelitis optica model induced by subarachnoid AQP4-IgG
Source: J Clin Invest. 2026 Feb 10;136(7):e199706. doi: 10.1172/JCI199706 (PMC13038209; doi:10.1172/JCI199706)
Supplement: Supplemental data [file jci-136-199706-s009.pdf]

Supplementary methods

3D image segmentation. Image segmentation, volume rendering and visualization were conducted using Reconstruct software packages (1). Neuron, microglia, and neutrophil morphology were identified in every 100-nm-thick section based on confocal imaging. For neutrophil segmentation, multi-lobed nuclear structure and types of membrane-bound cytoplasmic granules were the main neutrophil identifying characteristics (2). The neutrophil boundary was outlined based on darker cytoplasm compared to that of microglia. For microglial segmentation, we observed a relatively dark nucleus with clumped chromatin and lysosomes in lighter cytoplasm (3). Neurons were defined by clear nucleus with prominent nucleoli and a distinctive soma with abundant endoplasmic reticulum (4) .

CD68 imaging and analysis. Lumbar cord sections were incubated overnight at 4 °C with rabbit-CD68 (Abcam, ab125212, 1:500) and goat anti-IBA1 (Wako; 011-27991, 1:500), diluted in PBS containing 0.25% Triton X-100. Following primary antibody incubation, sections were washed three times in PBS (5 min each) and stained 2 hrs with Alexa 594 donkey anti-rabbit (1:1,000) and Alexa 488 donkey anti-goat IgG (1:1,000). Nuclei were counterstained with DAPI (Sigma, 0.1 µg/mL) and mounted with SlowFade™ Diamond Antifade Mountants (Thermo Fisher Scientific). The CD68 expression in the ventral grey matter was analyzed using a LSM980 confocal microscope with ×40 water objective lens (20 z slice, 0.5 µm per slice). CD68 fluorescence (excitation/emission: 561 nm/590-618 nm) and IBA1 fluorescence (488 nm/496-525 nm) were captured. Binary CD68 images were generated using a uniform threshold in ImageJ, and CD68 expression was quantified from maximum-intensity Z-projections. The percentage of CD68 expression was calculated as the $CD68^+$ area relative to the $IBA1^+$ area. 3D renderings of $CD68^+$ puncta, $IBA1^+$ microglia, and $DAPI^+$ nuclei were generated using the "Surface" model function in

Imaris v10.0, with transparency adjustments applied to microglial surface rendering for enhanced visualization and spatial analysis.

Sholl analysis using Imaris filament tracer. Microglial branch analysis of IBA1-stained lumbar anterior horn tissue was conducted using confocal Z-stack images acquired with a × 63 oil immersion lens (Zoom × 0.6, 2,048 × 2,048 pixels, 0.082 μm per pixel, 0.5 μm Z-step, 40 slices). Imaris software (v.10.0, Oxford Instruments) was utilized to render and quantify 3D microglial branches via the "Filament tracing" module (https://imaris.oxinst.com/versions/10). For analysis, we examined 3-5 randomly selected cropped 3D volumes per confocal image. The microglial soma diameter was set to 6 μm based on DAPI staining, and the minimum process detail was determined to be 0.35 μm. The tracing workflow included soma detection, seed point identification along cellular processes, and computation of filamentous segments. Segments were classified as "good" or "bad" by comparison with confocal images. To enhance efficiency and accuracy, we employed an AI-powered filament tracer integrated with an intensity-based segmentation approach to differentiate genuine microglial processes from background. A training set of ~100 manually annotated seed points per group were generated for machine learning-based classification, which was iteratively refined through an automated classification process. For process intersection quantification, we generated concentric spheres centered on the soma, increasing in 1 μm increments from a 6 μm radius. Intersection values at each radius were calculated using "Filament No. of Sholl intersections" model in Imaris.

Nissl staining and analysis. Lumbar cord sections were rehydrated in 1× PBS (pH 7.2) for 30 min, followed by permeabilization in 1× PBS containing 0.1% Triton X-100 for 10 min. Sections were then washed twice (5 min each) in 1× PBS. NeuroTrace staining (1:150 dilution in 1× PBS) was applied at room temperature for 20 min in a 96-well plate. After staining, sections were washed in

1× PBS containing 0.1% Triton X-100 for 10 min, followed by two additional 5-minute washes in 1× PBS at room temperature. Sections were washed overnight at 4 ℃, mounted and coverslipped using SlowFade™ Diamond Mountant (Thermo Fisher Scientific), and stored in the dark at room temperature to set the mounting medium. Images were acquired as Z-stack planes (3×4 tiling, 1,024 × 1,024-pixel resolution, 2.5 μm Z-steps, 4 slices) using a Zeiss LSM980 confocal microscope with ×10 objectives. Nissl count analysis in the lumbar ventral grey matter was performed using maximum-intensity confocal Z-stack images. A uniform threshold was applied to define the Nissl signal ROI. The number of Nissl-positive cells and Nissl signal area were quantified using the ImageJ "Analyze Particle" function (NIH, Bethesda, MD). The % Nissl area was calculated by normalizing total Nissl signal area to the ventral grey matter outline.

WT Mouse neutrophil culture and immunostaining. Pooled blood, spleen, and bone marrow was depleted of erythrocytes by lysis in ACK buffer. Neutrophils were isolated by gradient centrifugation (500 × *g* for 20 minutes) on 65% and 55% Percoll. Neutrophils collected from the pellet (PBMCs remained at the 65% and 55% Percoll interface) were plated on poly-D-lysine and laminin glass coverslips and stimulated for 4 hrs with 25 ng/mL TNF-α diluted in serum-free Gibco™ DMEM and 1% penicillin/streptomycin. Immunofluorescence staining was as described for mouse astrocytes, except with permeabilization in PBS containing 0.05% Triton X-100 extended to 30 mins and blocking with 3% donkey serum. To avoid endogenous IgG interference, mouse-on-mouse IgG blocking kit (Vector Laboratories, BMK-2202) was used for anti-mouse C5 labeling. Donkey anti-mouse IgG 647 F(ab')$_2$ secondary antibody (Abcam; ab181292, 1:1,1000) was applied to prevent Fcγ-mediated binding to immune cells following incubation with mouse anti-C5 IgG. Primary antibodies: goat anti-myeloperoxidase/MPO (R&D; AF3667; 1:500), Ly6G-PE (Biolegend, 127608, 1:100), mouse anti-C5 (Hycult Biotech; HM1073, 1:200), rat anti-C5a

(Invitrogen; MA5-23910, 1:200). Alexa Fluor-conjugated secondary antibodies: donkey anti-mouse IgG, 647, F(ab')$_2$, donkey anti-goat 488, and donkey anti-rat 650 were diluted in 1% BSA in PBS/0.05% Trion X-100 for 1 h at RT. Fluorescence and bright-field images were acquired using a Zeiss LSM980 confocal microscope at 63× magnification with a 3.8× digital zoom. High-resolution images for quantifying of C5 and C5a puncta within neutrophils were obtained using the Zeiss LSM980 Airyscan2 module with an oil immersion lens.

References

1. Fiala JC. Reconstruct: a free editor for serial section microscopy. *J Microsc.* 2005;218(Pt 1):52–61.
2. Brinkmann V, and Zychlinsky A. Neutrophil extracellular traps: is immunity the second function of chromatin? *J Cell Biol.* 2012;198(5):773–83.
3. Garaschuk O, and Verkhratsky A. Physiology of Microglia. *Methods Mol Biol.* 2019;2034:27–40.
4. Lee WC, Bonin V, Reed M, Graham BJ, Hood G, Glattfelder K, et al. Anatomy and function of an excitatory network in the visual cortex. *Nature.* 2016;532(7599):370–4.

Supplementary figures

Figure S1

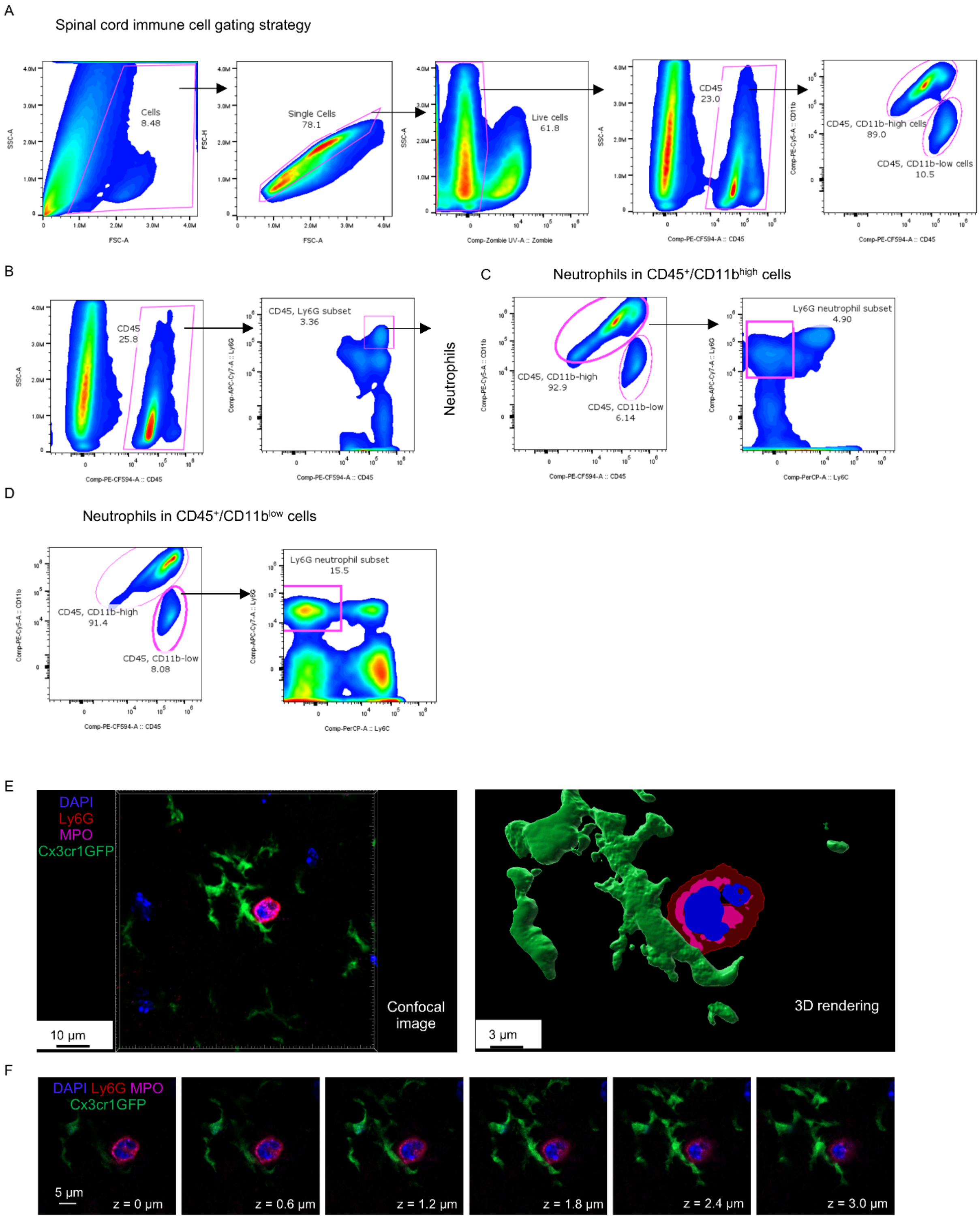

Figure S1. Gating strategies to identify neutrophils in spinal cord by Cytek Aurora system and nuclear segmentation in neutrophil. Related to Figure 1 and Figure 4

A-D. Flow cytometry gating strategies to investigate the frequency of $Ly6G^+$ neutrophils among $CD45^+$/$CD11b^+$ immune cells enzymatically dissociated from spinal cord tissues of indicated mice after transcardiac washout of vasculature.

E. Representative neutrophil ($Ly6G^+$, red) interacting with microglial processes ($Cx3cr1GFP^+$, green). nuclear segmentation ($DAPI^+$, blue) and granule protein (MPO^+, magenta) were observed in this neutrophil.

F. A series of Z-stack images corresponding to panel E show the colocalization of neutrophil membrane and microglial processes.

Figure S2

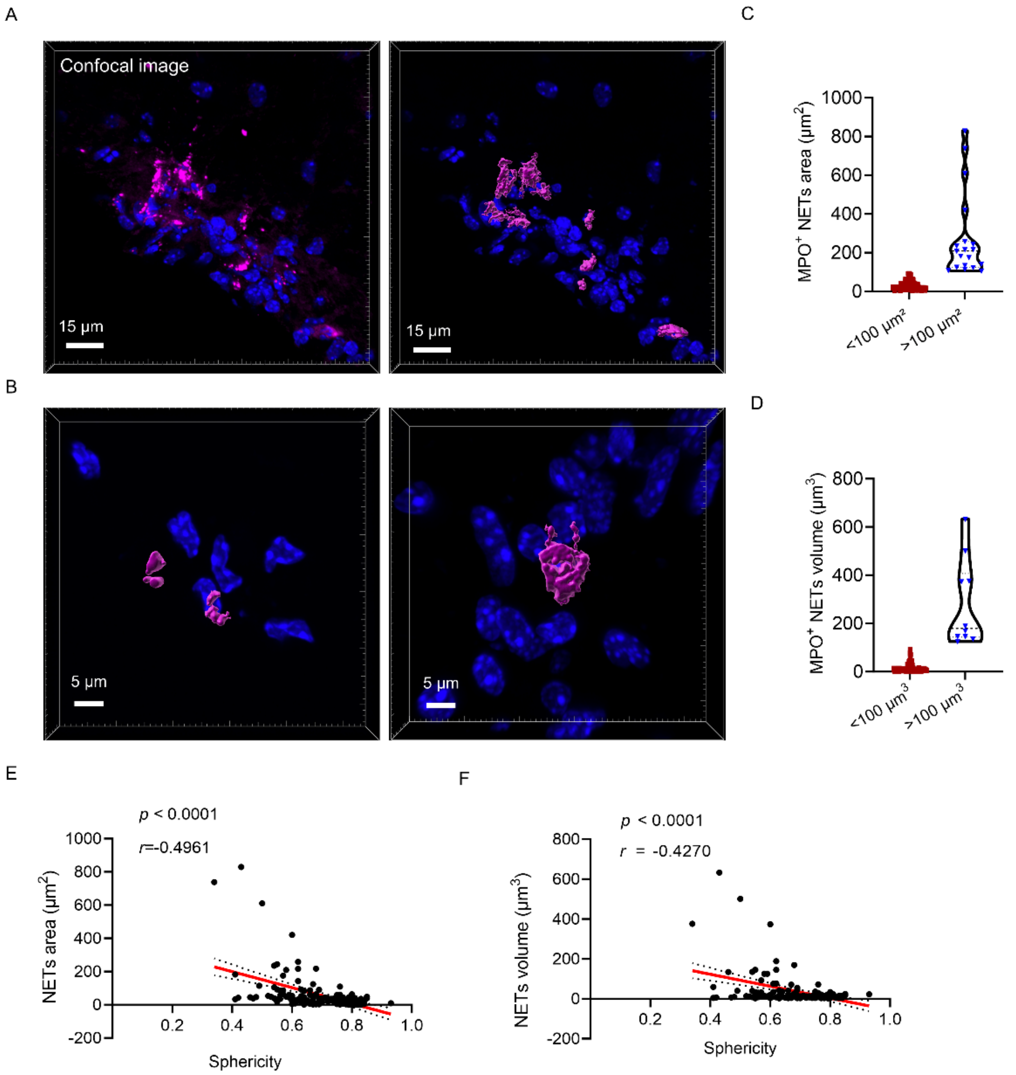

Figure S2. Morphological features of netting neutrophils in lumbar parenchyma in NMO mouse model

A. Left: Representative confocal image showing MPO⁺ neutrophil extracellular traps (NETs; magenta) with DAPI nuclear staining (blue) in the spinal cord at day 5 of AQP4-IgG infusion.

Right: 3D reconstruction of NETs generated using Imaris, revealing the characteristic web-like extracellular structures.

B. Representative 3D rendering showing various sizes of MPO^+ granule protein (left), granule protein extrusion (right) from an infiltrating neutrophil in the spinal cord.

C and D. Quantification of NETs area and volume, $n = 134$ NETs area < 100 μm^2, $n = 19$ NETs area > 100 μm^2; $n = 143$ NETs area volume < 100 μm^3; $n = 10$ NETs area volume > 100 μm^3.

E and F. Correlations between NETs morphology (area and volume) and NETs sphericity. Simple linear regression (one dot represents one NET particle at day 3 of AQP4-IgG infusion). In all graphs, data represent means ± SEM; all statistical tests are two-sided. Unpaired Student *t*-test in C and D, $p < 0.05$ was considered a significant difference.

Figure S3

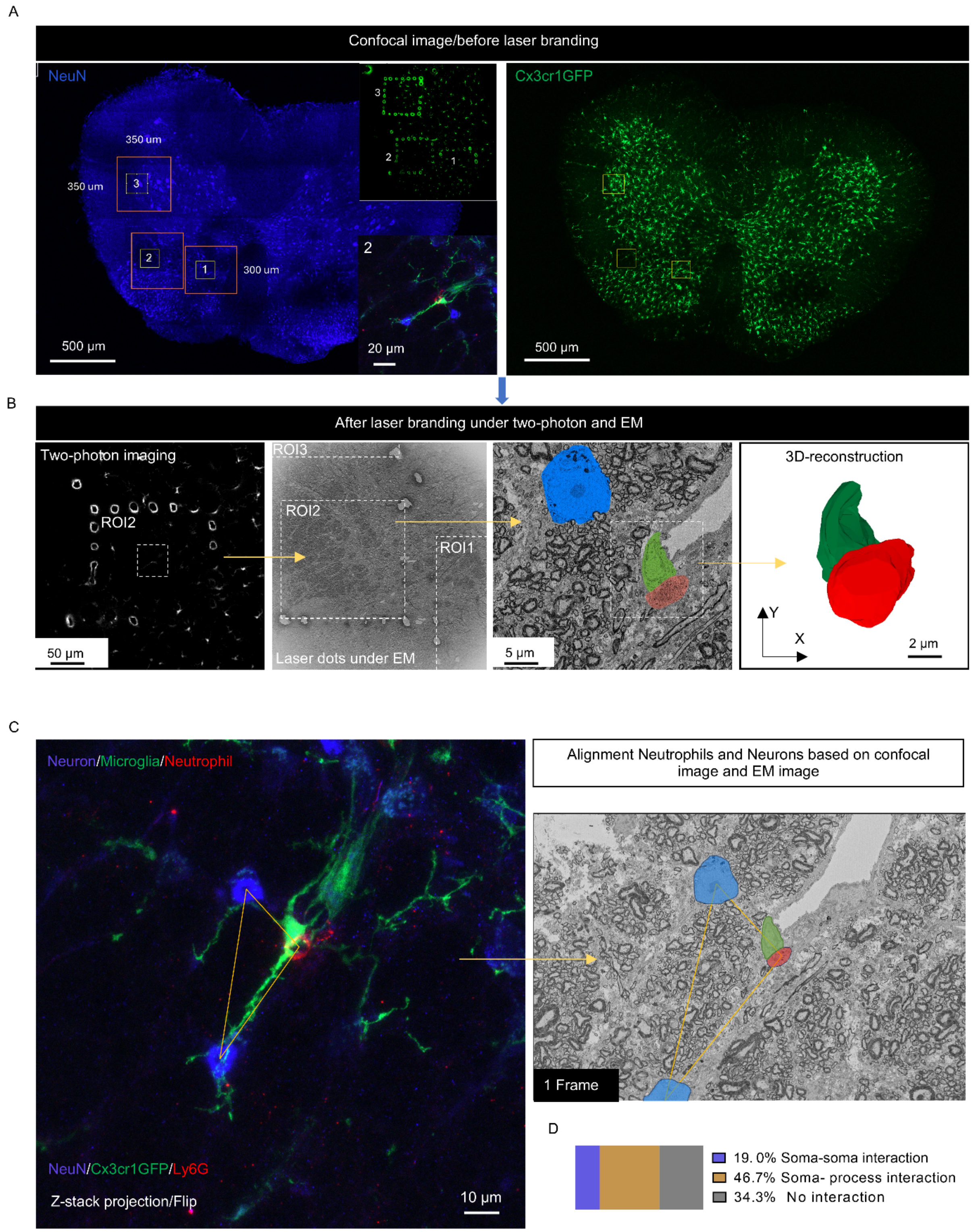
A
Confocal image/before laser branding
NeuN
350 um
350 um
3
2
1
300 um
500 μm
20 μm
Cx3cr1GFP
500 μm
B
After laser branding under two-photon and EM
Two-photon imaging
ROI2
50 μm
ROI3
ROI2
ROI1
Laser dots under EM
5 μm
3D-reconstruction
Y
X
2 μm
C
Neuron/Microglia/Neutrophil
NeuN/Cx3cr1GFP/Ly6G
Z-stack projection/Flip
10 μm
Alignment Neutrophils and Neurons based on confocal image and EM image
1 Frame
D
19. 0% Soma-soma interaction
46.7% Soma- process interaction
34.3% No interaction

Figure S3. Near-infrared branding by two-photon laser aligns confocal and electron microscopic images to confirm microglia-neutrophil interaction. Related to Figure 2

A. Regions of interest (ROI) were identified in a whole mount lumbar section by confocal microscopy before laser branding. $NeuN^+$ neurons (left) and $Cx3cr1GFP^+$ microglia (right) were imaged.

B. After laser branding, under 2-photon imaging, the region ROI2 (outlined by branding sites) was relocated by electron microscopy. The same interacting microglial-neutrophil pair was imaged by high resolution electron microscopy and subjected to 3D-serial reconstruction.

C. Alignment of neutrophils and neurons according to the confocal Z-stack images and electron microscopy images. The triangular pattern connecting cell somata indicated good alignment.

D. The percentage of each neutrophil interaction type with microglia.

Figure S4

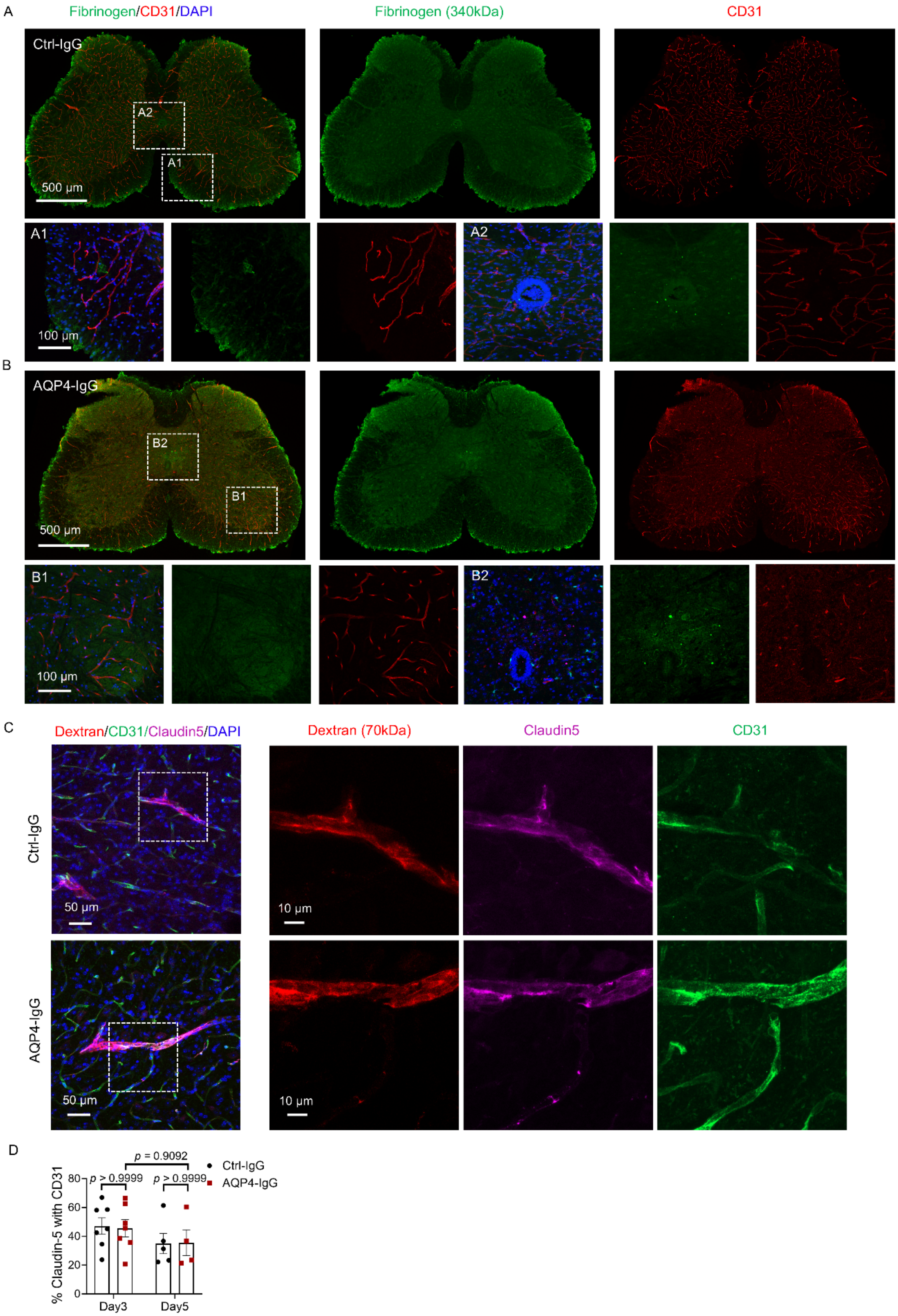
A
Fibrinogen/CD31/DAPI
Fibrinogen (340kDa)
CD31
Ctrl-IgG
A2
A1
500 µm
100 µm
B
AQP4-IgG
B2
B1
500 µm
100 µm
C
Dextran/CD31/Claudin5/DAPI
Dextran (70kDa)
Claudin5
CD31
Ctrl-IgG
AQP4-IgG
50 µm
10 µm
D
p = 0.9092
p > 0.9999
p > 0.9999
Ctrl-IgG
AQP4-IgG
% Claudin-5 with CD31
0
20
40
60
80
Day3
Day5

Figure S4. Blood-brain barrier integrity is preserved at day 3 in this NMO mouse model

A-B. Representative images of blood vessel ($CD31^+$, red) and fibrinogen (green) immunoreactivities in lumbar cord sections from Ctrl-IgG recipient mice (A) and AQP4-IgG recipient mice (B). Merged images, left. Boxed areas from Ctrl-IgG recipient mice are enlarged in A1 and A2. Boxed areas from AQP4-IgG recipients are enlarged in B1 and B2. C. Confocal images of blood vessel ($CD31^+$, green), Dextran red and Claudin 5 (magenta) immunoreactivities in lumbar cord sections from indicated mice. D. Quantification of the percentage area occupied by Claudin-5 of CD31 blood vessels in lumbar sections from day 3 and day 5 of AQP4-IgG and Ctrl-IgG infused mice (Treatment: $F_{(1, 19)} = 0.0055$, p=0.9417; time: $F_{(1, 19)} = 2.600$, p=0.1233; $n = 4$-7 mice per group).

Figure S5

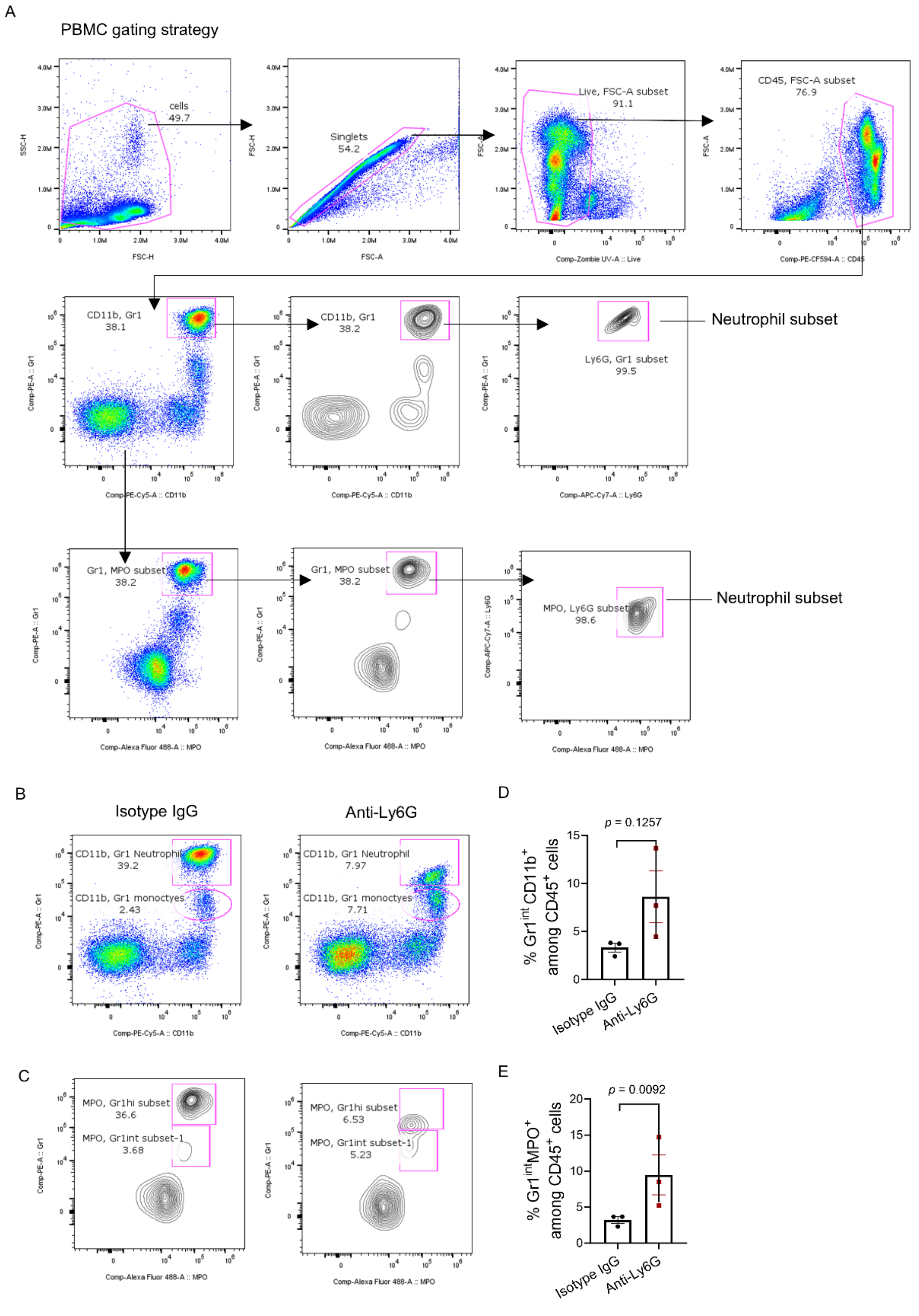
A
PBMC gating strategy
cells
49.7
Singlets
54.2
Live, FSC-A subset
91.1
CD45, FSC-A subset
76.9
CD11b, Gr1
38.1
CD11b, Gr1
38.2
Ly6G, Gr1 subset
99.5
Neutrophil subset
Gr1, MPO subset
38.2
Gr1, MPO subset
38.2
MPO, Ly6G subset
98.6
Neutrophil subset
B
Isotype IgG
Anti-Ly6G
CD11b, Gr1 Neutrophil
39.2
CD11b, Gr1 monocytes
2.43
CD11b, Gr1 Neutrophil
7.97
CD11b, Gr1 monocytes
7.71
C
MPO, Gr1hi subset
36.6
MPO, Gr1int subset-1
3.68
MPO, Gr1hi subset
6.53
MPO, Gr1int subset-1
5.23
D
p = 0.1257
% Gr1int CD11b+ among CD45+ cells
Isotype IgG
Anti-Ly6G
E
p = 0.0092
% Gr1intMPO+ among CD45+ cells
Isotype IgG
Anti-Ly6G

Figure S5. Gating strategies to assess neutrophil ablation efficiency in PBMC. Related to Figure 3

A. Flow cytometric gating strategies to determine the frequency of $Ly6G^{+}Gr1^{+}$ and $Ly6G^{+}MPO^{+}$ neutrophil subsets among peripheral immune cells.

B, C. Flow cytometric analysis was performed to identify the frequency of $Gr1^{int}$ $CD11b^{+}$ immature myeloid cells in B and $Gr1^{int}$ MPO^{+} immune neutrophils in C among peripheral immune cells.

D, E. Data summary in B and C.

Figure S6

A

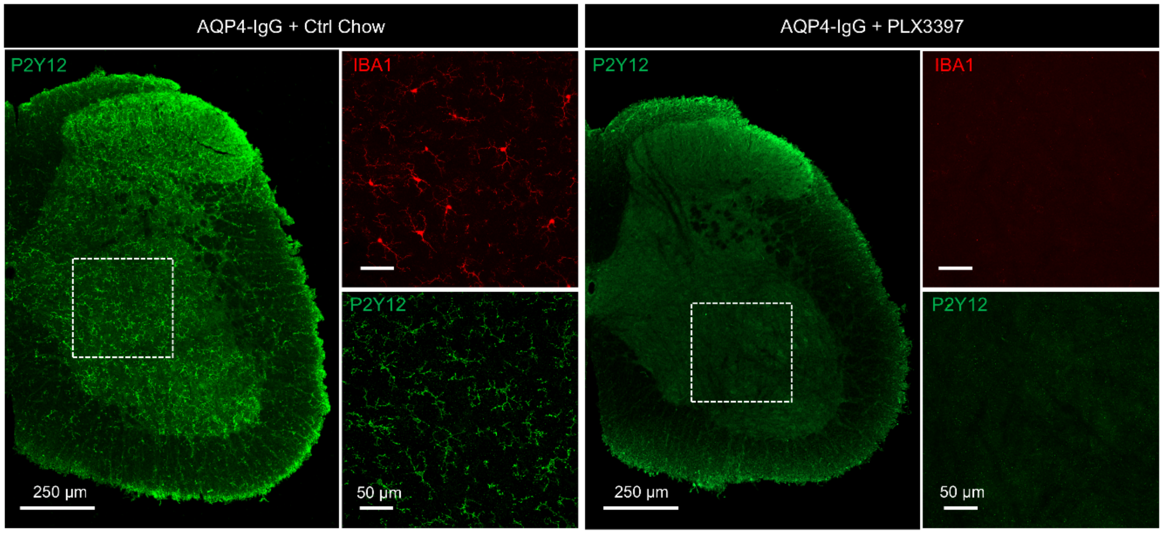

B

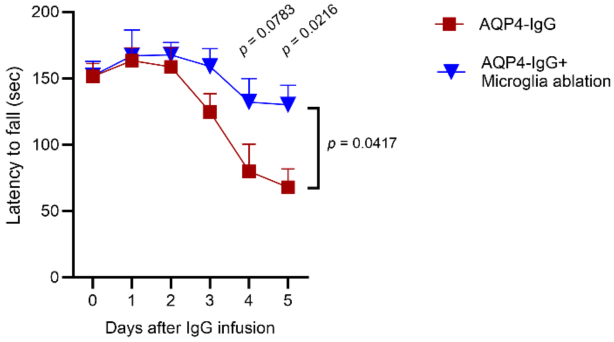

Figure S6. Motor impairment was ameliorated by microglial ablation in NMO mouse model

A. Representative images of P2Y12$^+$ (green) and IBA1$^+$ (red) microglia in L4 spinal cord sections of mice infused for 5 days with AQP4-IgG after continuous feeding of control chow or microglia-depleting PLX3397 chow starting 24 days before subarachnoid infusion.

B. Data for motor impairment, assessed by Rotarod testing, were analyzed by two-way (time × treatment) repeated ANOVA following Sidak multiple comparisons test using GraphPad Prism 8.

$F_{(1, 8)} = 5.865$, $p=0.0417$ for treatment (microglial ablation or non-ablation); $F_{(5, 40)} = 9.459$, $p < 0.0001$ for time; $n = 5$ mice per group.

Figure S7

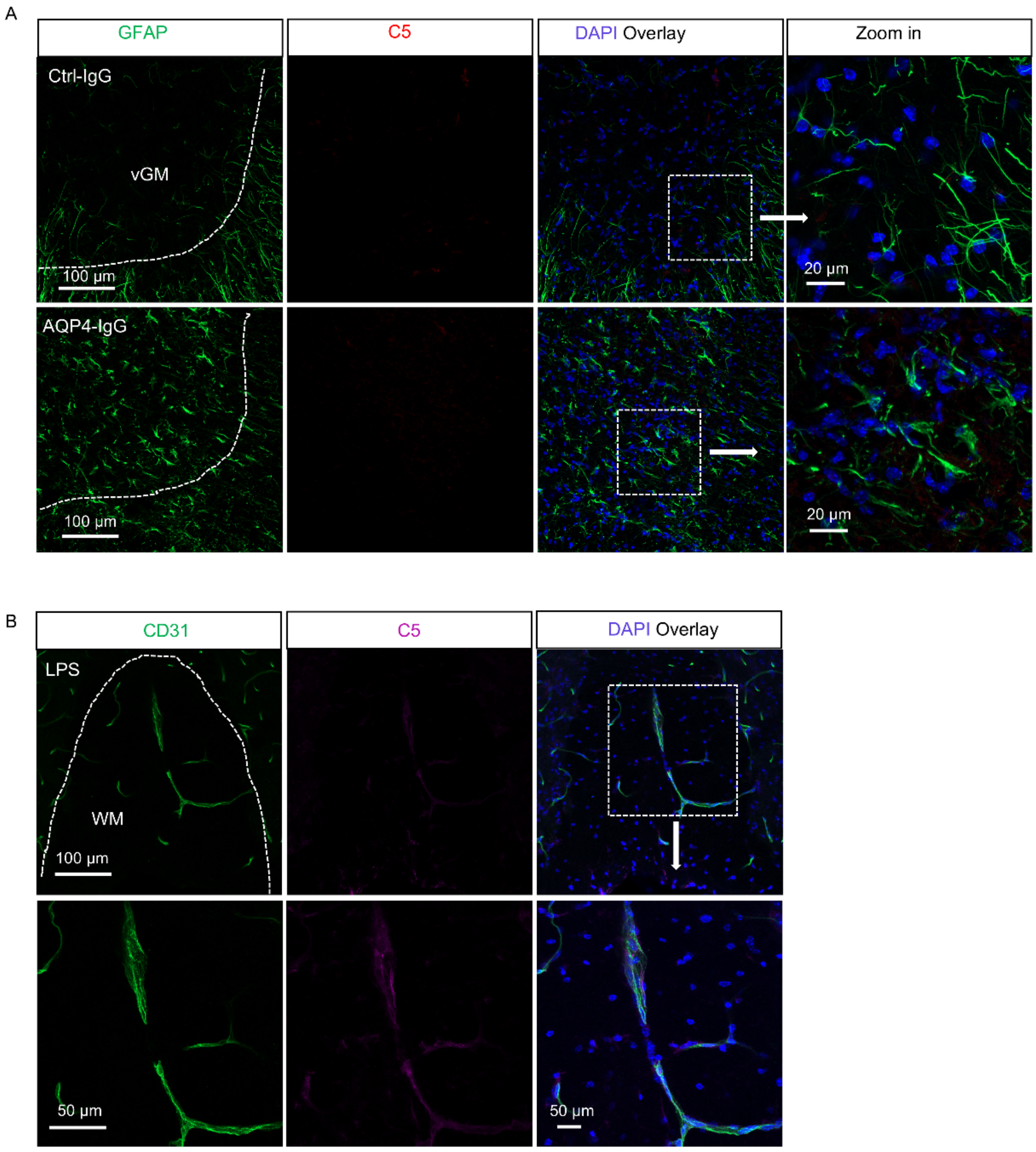

Figure S7. Astrocytes do not express C5 proenzyme in NMO mouse model. Related to Figure 4

A. No complement C5 immunoreactivity was detected in the cytoplasm of astrocytes ($GFAP^+$) or any other parenchymal resident or infiltrating cells in lumbar cord of mice infused with either Ctrl-IgG or AQP4-IgG. vGM: ventral grey matter.

B. Positive control image demonstrates C5-immunoreactivity within the lumen of a penetrating $CD31^+$ blood vessel in spinal white matter of a WT mouse injected *i.v.* with lipopolysaccharide (LPS, Sigma, L2880, 1.0 mg/kg, 50 µL).

Figure S8

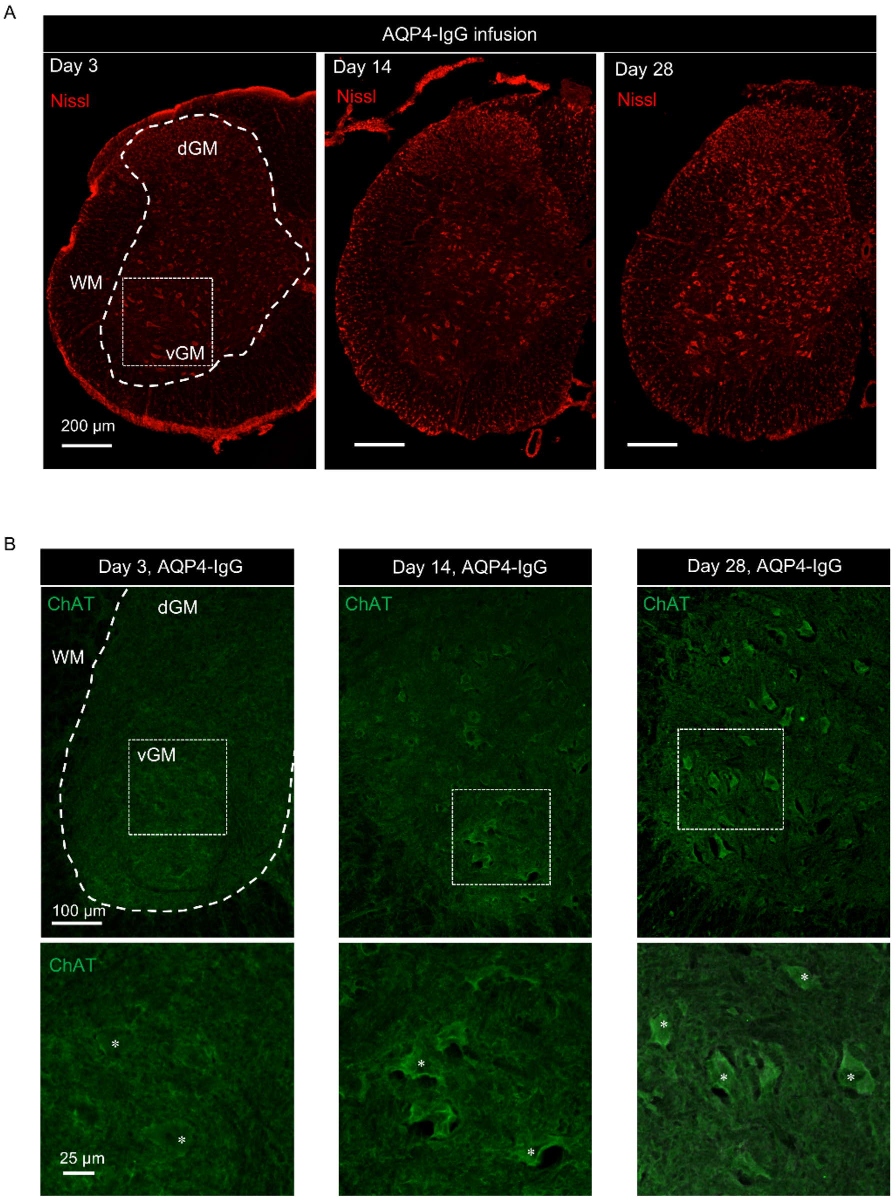

Figure S8. Neuronal dysfunction is reversible in this NMO mouse model. Related to Figure 6 B-D

A. Representative images of neuronal Nissl body staining in L4 cord sections of AQP4-IgG recipient mice at days 3, 14 and 28. AQP4-IgG infusion stopped at day 7. vGM: ventral grey

matter; dGM: dorsal grey matter; WM: white matter. B. Representative images of ChAT-immunoreactive motor neurons in L4 cord sections of AQP4-IgG recipient mice at days 3, 14 and 28. Asterisks indicate cytoplasmic ChAT-immunoreactivity, which is less intense at day 3 (lower).

Figure S9

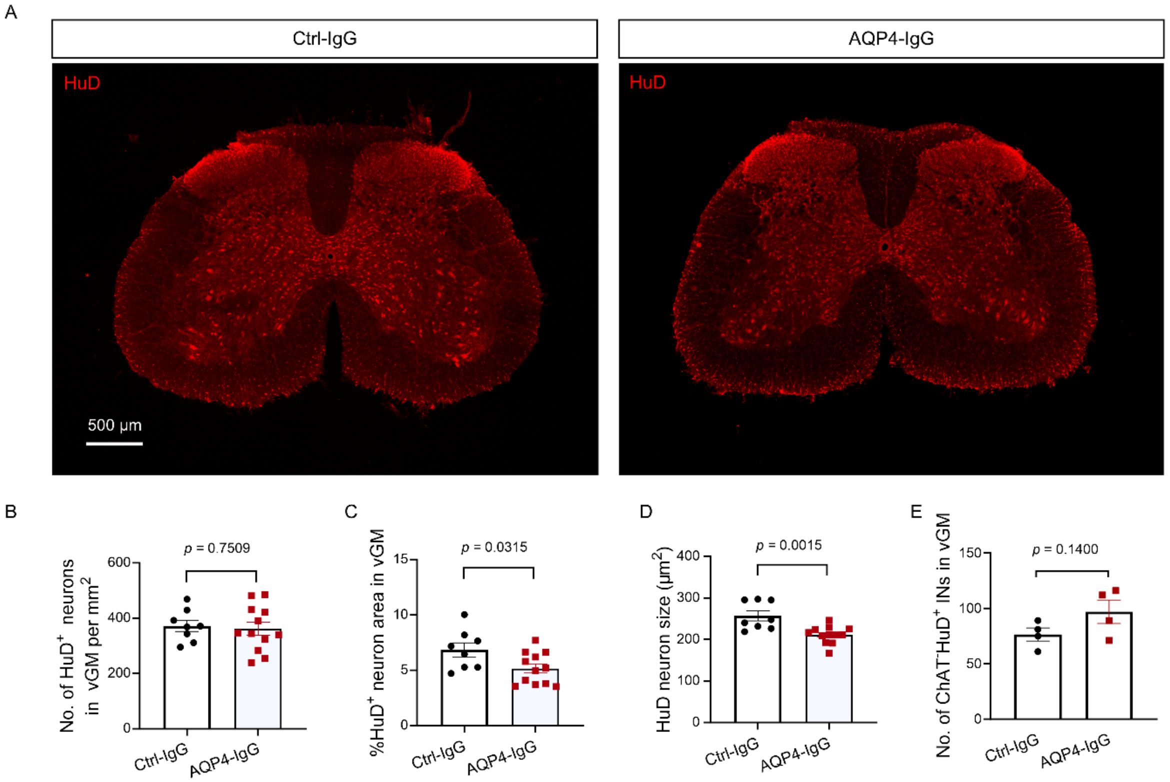

Figure S9. Neuronal changes consistent with functional impairment in this NMO mouse model

A. Confocal images show HuD-immunoreactivity (RNA-binding protein antigen of ANNA1-IgG) in neurons within representative L4 cord sections of mice infused by subarachnoid route with Ctrl-IgG or mouse monoclonal AQP4-IgG. (n = 8-12 sections from 4-6 mice per group).

B-D. The numbers of HuD^+ neurons (B); the percentage of HuD^+ neuron area (C) and HuD^+ neuronal average size (μm^2) (D) in the vGM of L4 cord. (n = 8-12 sections from 4-6 mice per group). vGM: ventral grey matter. E. Quantification of the number of $ChAT^-HuD^+$ interneurons in the vGM of L4 cord. (n = 4 mice per group). Data represent means ± SEM and all statistical tests are two-sided. Unpaired Student *t*-test. $p < 0.05$ was considered a significant difference.

Figure S10

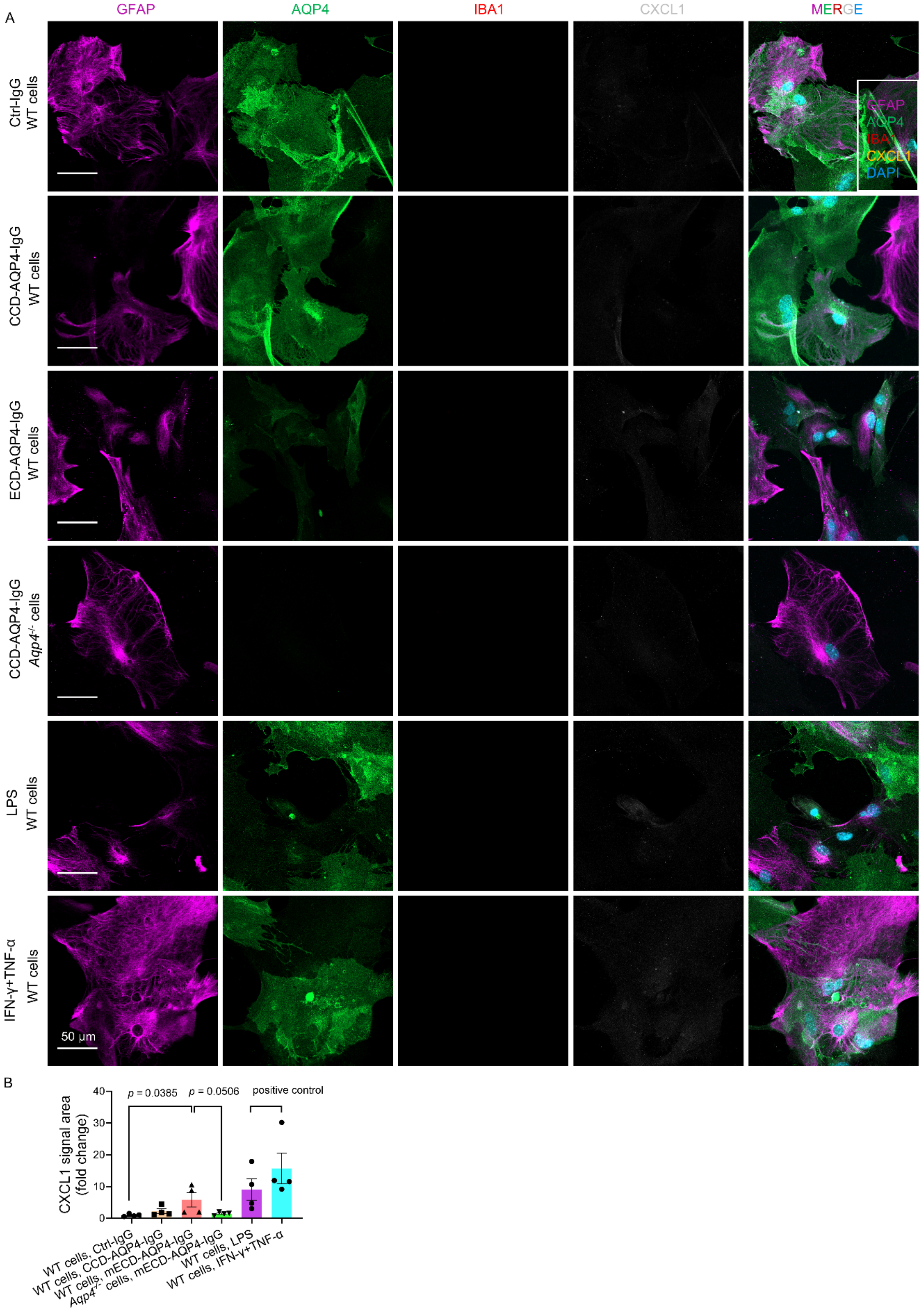
A
GFAP
AQP4
IBA1
CXCL1
MERGE
Ctrl-IgG WT cells
CCD-AQP4-IgG WT cells
ECD-AQP4-IgG WT cells
CCD-AQP4-IgG Aqp4-/- cells
LPS WT cells
IFN-γ+TNF-α WT cells
GFAP
AQP4
IBA1
CXCL1
DAPI
50 µm
B
CXCL1 signal area (fold change)
40
30
20
10
0
p = 0.0385
p = 0.0506
positive control
WT cells, Ctrl-IgG
WT cells, CCD-AQP4-IgG
WT cells, mECD-AQP4-IgG
Aqp4-/- cells, mECD-AQP4-IgG
WT cells, LPS
WT cells, IFN-γ+TNF-α

Figure S10. Upregulation of astrocytic CXCL1 production in response to pathogenic AQP4-IgG is greatly diminished in mouse glial cultures depleted of microglia

A. Microglia were depleted from primary cultures of neonatal mouse glia by adding Clodrosome (100 µg/mL; Encapsula Nano Sciences LLC) for 72 hrs. No microglial immunostaining (IBA1) was detected. Astrocytic cytoplasm is identified by GFAP immunostaining; AQP4-immunoreactivity was abundant on astrocytes exposed to control mouse IgG or a non-pathogenic monoclonal mouse IgG specific for the AQP4 cytoplasmic C-terminal domain (mCCD; negative control) but was largely cleared by endocytosis-degradation induced by exposure to the pathogenic monoclonal mouse IgG specific for the AQP4 extracellular domain (mECD) and is not expressed on astrocytes derived from *Aqp4*$^{-/-}$ mice. B. CXCL1 signal intensity was quantified from A images. Data represent means ± SEM and all statistical tests are two-sided. ($n = 4$ wells). One-way ANOVA with Tukey *post hoc* and unpaired Student *t* test in A. $p < 0.05$ was considered a significant difference.

Movie S1. Tracing of neuron (NeuN$^+$, blue), microglia (Cx3cr1GPF$^+$, green), and neutrophil (Ly6G$^+$, red) based on serial electron microscopic images, related to Figure 2C.

Movies S2. Interacting microglia (Cx3cr1GFP$^+$, transparent) and neutrophil (Ly6G$^+$); video created by Animation function in Imaris based on confocal Z-stack images, related to Figure 2E.

Movie S3. Interacting microglia (IBA1$^+$) and netting neutrophil (MPO$^+$); nuclear, blue (DAPI); video created by Animation function in Imaris based on confocal Z-stack images, related to Figure 2F.

Movie S4. Behavioral video showing Rotarod motor performance of mice infused with Ctrl-IgG (left) or pathogenic AQP4-IgG (right).

Movie S5. Behavioral video showing Rotarod motor performance of mice infused with AQP4-IgG alone (right 2 mice) or combined with anti-CXCL1-IgG (left 3 mice).
