# Supplementary material for: Neutrophil-microglia interaction drives motor dysfunction in a neuromyelitis optica model induced by subarachnoid AQP4-IgG
Source: J Clin Invest. 2026 Feb 10;136(7):e199706. doi: 10.1172/JCI199706 (PMC13038209; doi:10.1172/JCI199706)

Unedited blot and gel images, relate to Fig. 7B

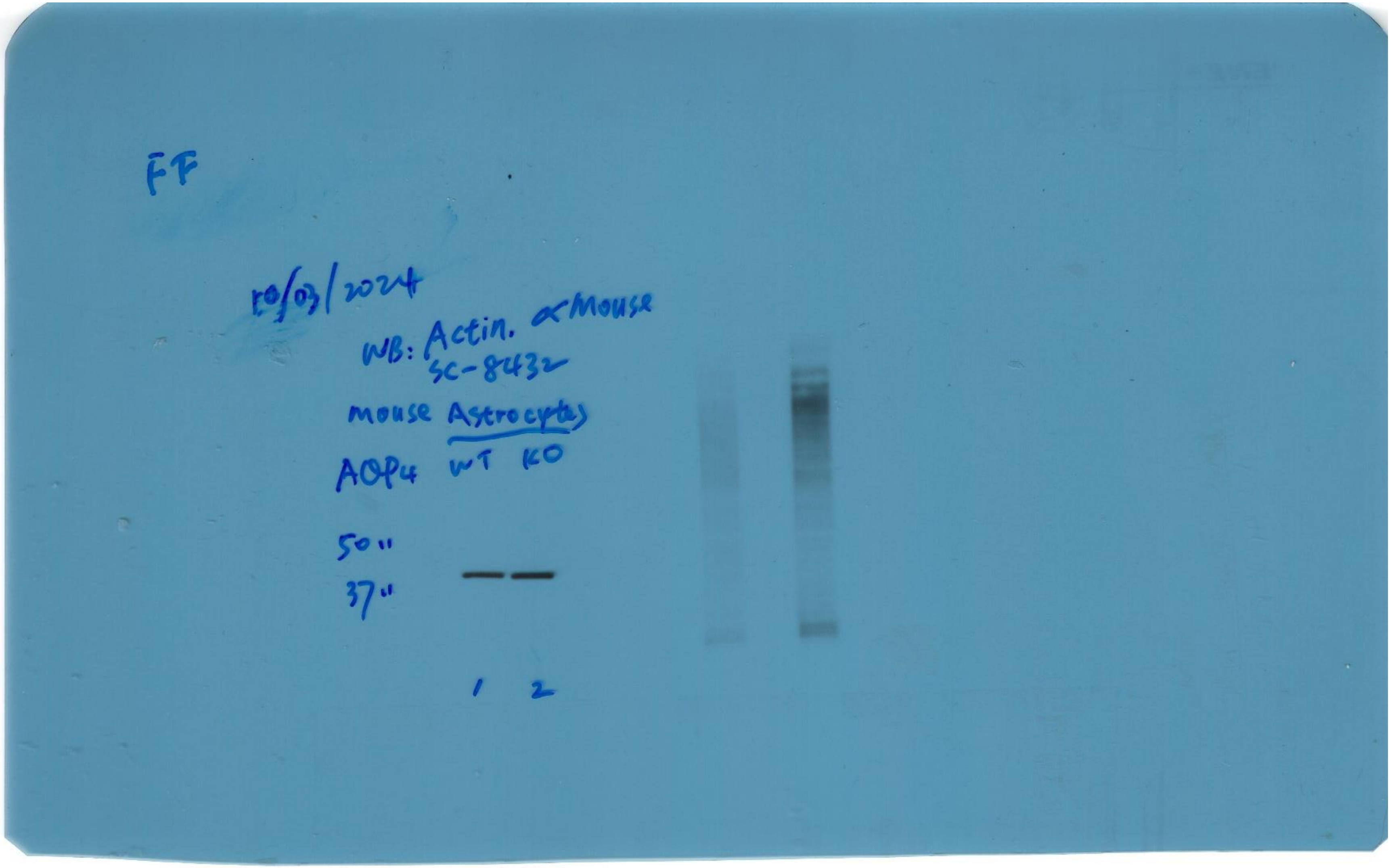

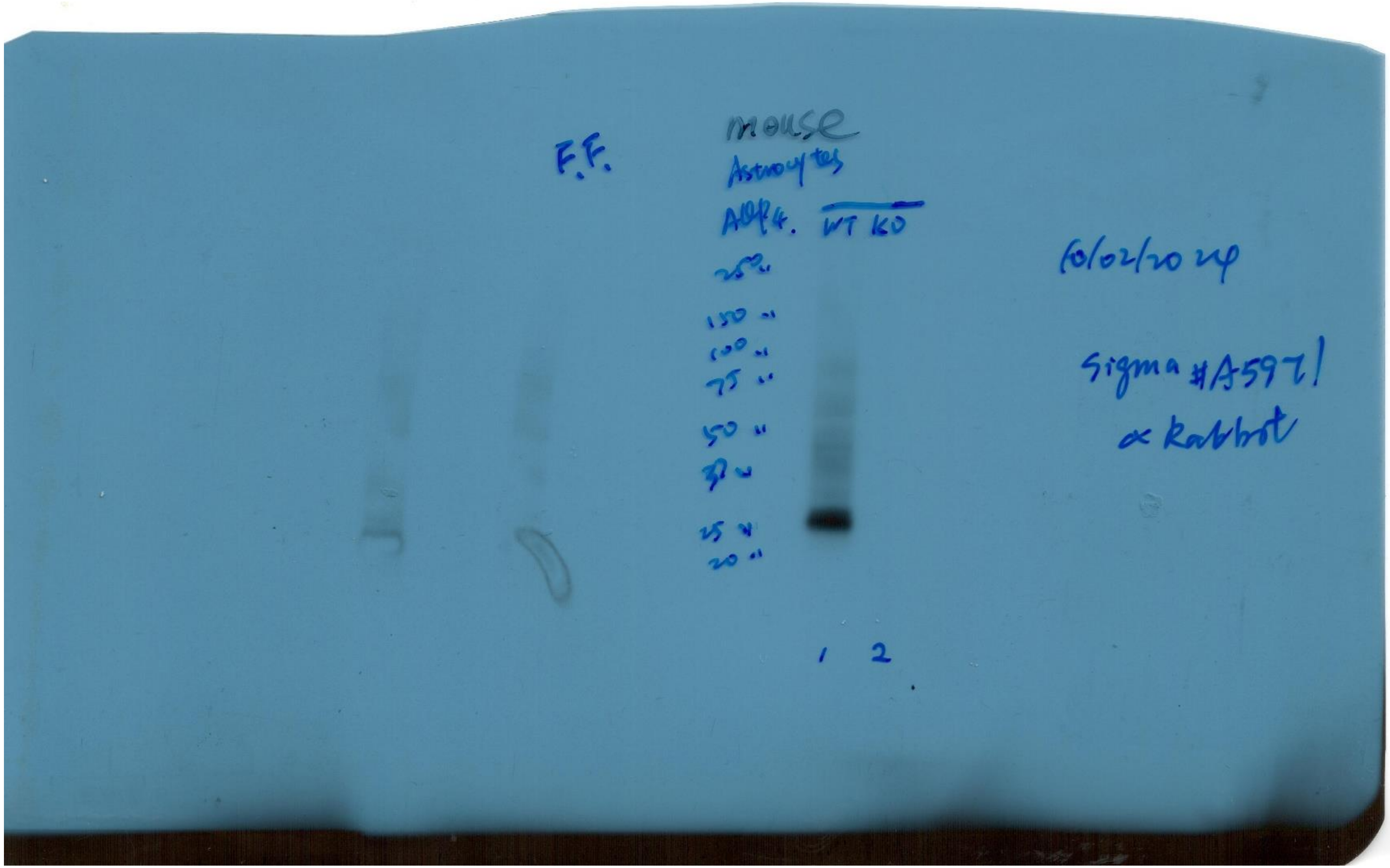

F.F.
mouse
Astrocytes
AQP4.
WT KO
10/02/2024
250
150
100
75
50
37
25
20
Sigma #A5971
α Rabbit
1 2

Fig. 7B

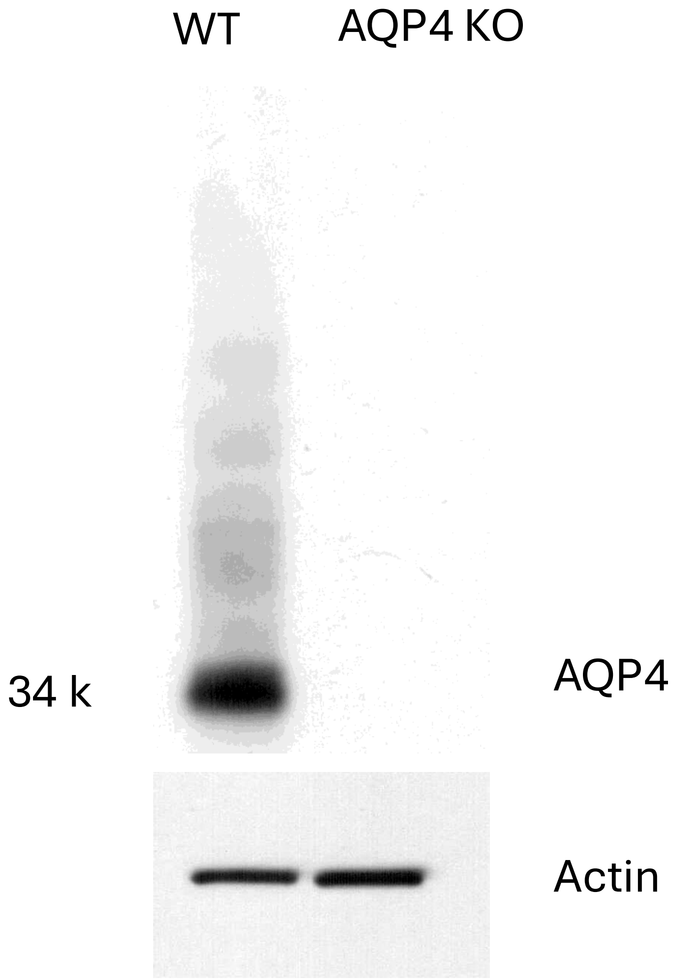

Supplement: Unedited blot and gel images [file jci-136-199706-s010.pdf]
